# Platelet-derived microparticles induce CD8^+^ T cell senescence in chronic kidney disease via the transfer of microRNA-550a-5p

**DOI:** 10.1038/s12276-026-01876-z

**Published:** 2026-10-16

**Authors:** Yuewen Sun, Qigang Lan, Yaqin Wang, Minhua Xie, Xinyi Wu, Liangjing Lv, Qing Xin, Jun Chen, Xiaoyi Zhong, Mengying Yao, Shuiqin Gong, Siyan Zhou, Junping Wang, Jinghong Zhao

**Affiliations:** 1 Department of Nephrology, Chongqing Key Laboratory of Prevention and Treatment of Kidney Disease, Chongqing Clinical Research Center of Kidney and Urology Diseases, Xinqiao Hospital, Army Medical University (Third Military Medical University), Chongqing, 400037 China; 2 Department of Nephrology, General Hospital of Western Theater Command, PLA, Chengdu, China; 3 State Key Laboratory of Trauma and Chemical Poisoning, Institute of Combined Injury, Chongqing Engineering Research Center for Nanomedicine, College of Preventive Medicine, Army Medical University (Third Military Medical University), Chongqing, 400038 China

**Keywords:** Senescence, Mechanisms of disease, CD8-positive T cells, Chronic kidney disease

## Abstract

Infection is a leading cause of mortality in patients with chronic kidney disease (CKD), in which T cell dysfunction is considered a typical feature. Although platelets are active participants in immune response, their role in regulating T cell function in patients with CKD remains unclear. In this study, we demonstrate that CD8^+^ T cell senescence may be a major contributor to the immunosuppressed state of patients with CKD, and that platelets derived from patients with CKD can induce premature CD8^+^ T cell senescence, potentially through mediating mitochondrial dysfunction. Further investigations reveal that platelet-derived microparticles (PMPs) from patients with CKD promote CD8^+^ T cell senescence via transferring miR-550a-5p, thereby decreasing PMPCB expression and inducing NDUFS8 cleavage failure. Finally, inhibition of miR-550a-5p and pretreatment with nicotinamide mononucleotide are capable of preventing platelet-induced CD8^+^ T cell senescence in CKD patients. Collectively, these findings suggest that the ectopic transient expression of platelet-derived miR-550a-5p in CD8^+^ T cells promotes cellular senescence by regulating the PMPCB–NDUFS8 axis, which can be exploited to treat CKD-associated immunosenescence.

**Figure:**
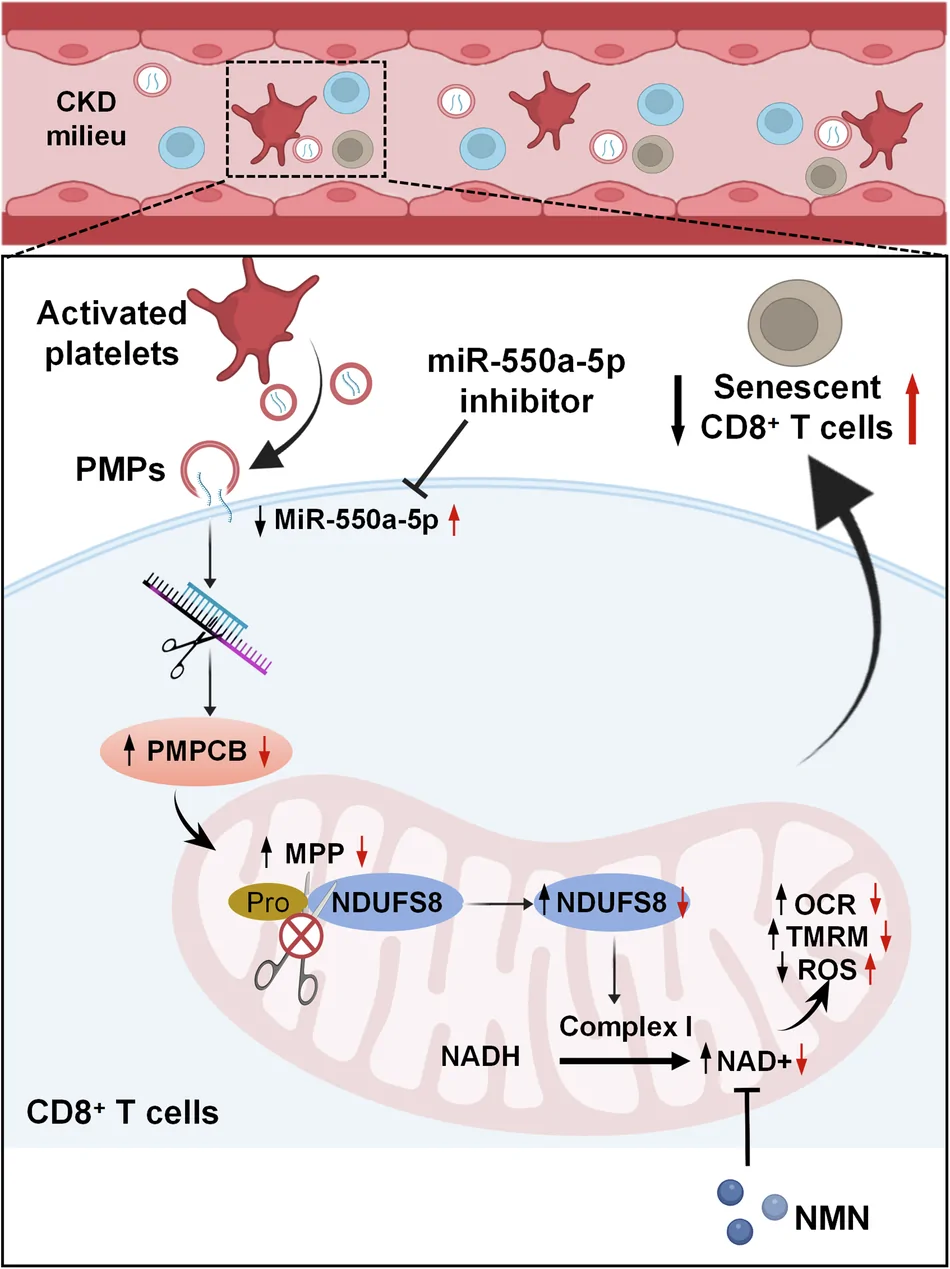

## Platelet Microparticles Drive CD8 T Cell Senescence in Chronic Kidney Disease

Chronic kidney disease (CKD) poses a significant global health challenge, with infection being a major cause of morbidity and mortality owing to immune dysfunction. This study reveals that premature senescence of CD8^+^ T cells is a key feature of immune dysregulation in patients with CKD. Researchers investigated the role of platelets in this process, showing that platelets from patients with CKD release microparticles (PMPs) containing miR-550a-5p, which induce mitochondrial dysfunction and senescence in CD8^+^ T cells by downregulating PMPCB and affecting the NDUFS8/NAD^+^ axis. Combining human samples and animal models, the study demonstrated that inhibiting miR-550a-5p or overexpressing PMPCB could mitigate these effects. These findings highlight a novel mechanism of immune dysfunction in CKD and suggest potential therapeutic strategies, such as targeting the PMPCB–NDUFS8 axis or using nicotinamide mononucleotide to prevent CD8^+^ T cell senescence.

This summary was initially drafted using artificial intelligence, then revised and fact-checked by the author.

## Introduction

Chronic kidney disease (CKD) has emerged as a critical global public health concern and is accompanied by a high incidence of multiple complications^1,2^. It has been well recognized that infection is a leading cause of morbidity and mortality in patients with CKD, particularly those with end-stage renal disease (ESRD)^3^. Notably, the immunosuppressed state of patients with CKD is characterized by dysfunction of an innate and adaptive immune system, resulting in a proinflammatory milieu and decreased cellular immunity^4^. Unfortunately, understanding of the susceptibility to infection and immune dysfunction in patients with CKD is still limited, and more effective measures are still needed in clinical practice.

The adaptive immune system possesses highly specific features in response to threats from pathogen and memory function^5^. T cells are the major cell components and responsible for mediating cell-based immune responses^6^. In CKD, there is a profound change in the total number and function of circulating T cells^7,8^. Plenty of studies, including our own, have demonstrated a decrease in T cell number in CKD, as well as a concomitant increase in terminal differentiation and proinflammatory T cells^9,10^. Furthermore, in the presence of numerous stimulations, such as uraemia and dialysis, T cell replicative capacity is impaired, resulting in functional exhaustion and senescence^11,12^. These two states share overlapping phenotypic and functional characteristics but differ in their distinct molecular and developmental signatures^13^. T cell exhaustion is marked by increased expression of a suite of inhibitory receptors, including PD-1 and cytotoxic T-lymphocyte antigen-4 (CTLA-4)^14^, whereas T cell senescence is defined by features such as telomere shortening and loss of CD28, along with reciprocal expression of CD57 (ref.^15^). However, the underlying mechanisms by which T cell exhaustion and senescence contribute to immune dysfunction upon CKD remain largely unexplored.

Platelets, one of the most prevalent blood components, are well-known for their haemostatic and thrombotic functions. Interestingly, accumulating evidence now indicates that platelets also actively modulate immune responses^16,17^. It is becoming increasingly clear that platelets can not only directly interact with immune cells by changing their surface expression of P-selectin or other receptors, such as Toll-like receptors but can also release a number of granules and microparticles that regulate adaptive immunity^18^. Our findings have also revealed disrupted platelet homeostasis in the CKD milieu, with enhanced platelet–immune cell interactions and increased release of platelet-derived microparticles (PMPs)^19,20^. Given that plasma microparticles can alter T cell function and phenotype, and that PMPs are the most abundant microparticles present in the blood^21^, it is important to improve our understanding of the role of platelet–T cell interactions in immune dysregulation upon CKD.

In this study, we demonstrate that platelets from CKD patients transfer miR-550a-5p through the release of PMPs, thereby inducing mitochondrial dysfunction-mediated cellular senescence via peptidase-mitochondrial processing subunit beta (PMPCB)–NADH: ubiquinone oxidoreductase core subunit S8 (NDUFS8) axis. Our findings uncover a novel platelet–CD8^+^ T cell interaction and provide a promising strategy for preventing CD8^+^ T cell senescence in CKD.

## Materials and methods

### Study subjects

The study was approved by the Ethics Committee of Xinqiao Hospital, Army Medical University (No. 2025-267-01). Only patients with primary nephropathy were included; those with secondary nephropathies, diabetes, recent immunosuppressant use (past 6 months), infections of any aetiology, or who were CMV-seropositive, were excluded (The CMV IgG screening results are presented in Supplementary Table 1). The participant recruitment process is shown in Supplementary Fig. 1. A total of 75 non-dialysed patients with CKD stage 3–5 (25–45 years old) were enrolled from the Department of Nephrology, Xinqiao Hospital (Chongqing, China), along with 45 healthy volunteers. For the phenotypic analysis, 30 patients with CKD stages 3–5 and 30 healthy controls were included. For the senescent degree analysis, an additional 45 patients with CKD stages 3–5 and 15 healthy controls were included. Subsequent mechanistic analyses were mainly performed using samples from 30 patients with CKD stage 5 and 30 healthy controls. Baseline characteristics are shown in Supplementary Table 2.

### Animal experiments

MiR-550a-5p agomir and negative control were synthesized by Biomics (The sequences are listed in Supplementary Table 3). Male C57BL/6J mice (8 weeks old) were purchased from Chongqing Tengxin Bioscience (Chongqing, China). Mice received tail vein intravenous injections of miR-550a-5p agomir or the negative control at a dose of 20 nmol once every 4 days for 4 weeks. All mice were euthanized 48 h after the final injection. Separately, mice were administered 100 µg of DiO-PMPs or saline via the tail vein and euthanized 48 h after injection. All the experimental procedures were approved by the Animal Care and Use Committee of the Army Medical University (No. AMUWEC20255414).

### Cell culture

HEK-293T cells were cultured with DMEM supplemented with 10% FBS and 1% penicillin/streptomycin. MEG-01 cells were cultured with RPMI 1640 medium supplemented with 10% FBS and 1% penicillin/streptomycin. To mimic the uraemic and inflammatory microenvironment, MEG-01 cells were incubated with 10% serum from patients with CKD. To mimic a chronic inflammatory environment, MEG-01 cells were co-cultured with peripheral blood mononuclear cells (PBMCs) derived from patients with CKD at a 1:10 ratio^22,23^.

### Isolation of human CD8^+^ T cells and platelets

Human whole blood was collected using sodium citrate anticoagulant. CD8^+^ T cells were isolated using the ImunoSep Human CD8^+^ T Cell Enrichment Kit (Precision Biomedicals, Shenzhen, China). The purity of the isolated CD8^+^ T cells, assessed by flow cytometry, exceeded 95%. Purified cells were resuspended in RPMI 1640 medium (Meilunbio, Dalian, China) supplemented with 10% FBS (Corning, NY, USA).

Platelet isolation was performed as previously described^20^. Briefly, blood was centrifuged at 160 *g* for 15 min at room temperature. The upper two-thirds of the platelet-rich plasma (PRP) were collected and then recentrifuged at 160 *g* for 10 min in the presence of 0.1 µg/ml prostacyclin. The resulting PRP was then centrifuged at 1,000 *g* for 10 min. The platelet pellet was resuspended in Tyrode’s-HEPES buffer containing 10 µmol/l indomethacin and 0.1 µg/ml prostacyclin. Leukocyte contamination in the platelet suspension was assessed by flow cytometric analysis of CD45^+^ events and was confirmed to be less than 0.001%.

### Isolation of platelet-derived microparticles

PMPs were isolated as previously described^24^. Purified platelets were resuspended in saline with 1 mM calcium and 1 U/ml thrombin at 37°C for 60 min. Activation was terminated by adding EDTA to a final concentration of 20 mM, followed by centrifugation at 2000 *g* for 20 min to remove platelets. The supernatants containing PMPs were centrifuged at 20,000 *g* for 90 min at 4°C. The protein concentrations of PMPs were determined by BCA protein assay. The size distribution of PMPs was analysed by dynamic light scattering using a Zetasizer Nano ZSE system (Malvern). PMP morphology was examined using a transmission electron microscope^25^.

### Co-culture with platelets or platelet-derived microparticles

CD8^+^ T cells were co-cultured with platelets as previously described^16^. Purified CD8^+^ T cells and platelets were co-cultured at a ratio of 1:450 in prepared RPMI 1640 medium for 7 days, with CD8^+^ T cells seeded at a concentration of 1 × 10^6^ cells/ml. T cell activation was induced using coated anti-CD3 and soluble anti-CD28 monoclonal antibodies (all Kxbiotech, Beijing, China). IL-2 (Beyotime, Shanghai, China) was added to maintain cell expansion. For T cell–PMP co-culture, CD8^+^ T cells were seeded at a concentration of 1 × 10^6^ cells/ml with PMPs (100 μg/ml) under the same culture conditions.

### Flow cytometry and cell sorting

Platelets were identified based on scatter properties. PMPs were detected as particles < 1 μm, and were identified by positive staining for CD41 and Annexin V, as well as negative staining for CD45 to exclude leukocyte contamination (all Thermo Fisher Scientific, MA, USA)^19^. Surface staining for CD61 and CD62P was performed to further characterize platelet origin. Platelet activation was assessed by measuring surface expression of P-selectin and activated integrin GPIIb/IIIa (both Thermo Fisher Scientific).

To determine T cell phenotype in peripheral blood, 100 µl of whole blood was processed to isolate PBMCs. For human T cells, PBMCs were stained with a lineage antibody cocktail comprising anti-CD3, anti-CD4, anti-CD8, anti-CD57, anti-PD-1 and anti-CD28 antibodies. For mouse T cells, a cocktail containing anti-CD3, anti-CD8, anti-CD44 and anti-CD153 antibodies was used.

To determine mitochondrial membrane potential, cells were incubated with 100 nM tetramethylrhodamine methyl ester (TMRM; Thermo Fisher Scientific) at 37°C for 20 min. To determine reactive oxygen species (ROS) production, cells were incubated with 10 μM dichlorodihydrofluorescein diacetate (DCFH-DA; Sigma-Aldrich, St. Louis, MO, USA) at 37°C for 30 min. Senescence-associated β-galactosidase (SA-β-Gal) activity was evaluated using the CellEvent Senescence Green Flow Cytometry Assay Kit (Thermo Fisher Scientific) according to the manufacturer’s protocol. To determine p21 and γ-H2A.X levels, cells were washed and fixed, and then resuspended with 1 ml Foxp3 Fixation/Permeabilization working solution (Thermo Fisher Scientific) and incubated at room temperature for 30 min. Then, the cells were permeabilized with Permeabilization buffer (Thermo Fisher Scientific) in the presence of anti-phospho-H2A.X (Ser19) and anti-p21 at room temperature for another 30 min.

For sorting of human CD57^+^/CD57^−^CD8^+^ or mouse CD8^+^ T cells, PBMCs were stained with indicative antibodies and isolated by fluorescence-activated cell sorting (FACS).

Cells were detected using a BD FACSverse flow cytometer (BD Biosciences) or sorted using a FACSAriaII (BD Biosciences). Data analysis was performed using FlowJo software (Tree Star Inc.).

Detailed information on those antibodies is listed in Supplementary Table 4.

### Relative telomere length measurement

To determine telomere length in CD8^+^ T cells, genomic DNA was extracted from isolated CD8^+^ T cells using the Fast DNA Extraction Kit (Bioroyee, Beijing, China) according to the manufacturer’s protocol. Relative telomere length was determined using the Relative Telomere Length Quantification qPCR Assay Kit (ScienCell Research Laboratories, San Diego, CA, USA).

### Cell mito stress test

Oxygen consumption rate (OCR) was measured using the Seahorse XF HS Mini Analyzer (Agilent Technologies, Santa Clara, CA, USA). CD8^+^ T cells were seeded in XF8 cell-culture microplates and analysed with the Seahorse XFp Cell Mito Stress Test Kit (Agilent Technologies). Following three baseline respiration measurements without additives, sequential injections of mitochondrial modulators were performed: 1.5 μM oligomycin (ATP synthase inhibitor), by the addition of 2 µM FCCP and rotenone and antimycin A (0.5 µM Rot/AA) for further measurements. OCR was expressed as units of picomoles (pmol) per minute, and data were normalized against the number of cells.

### NAD^+^/NADH assay

NAD^+^/NADH levels were quantified using the NAD^+^/NADH Assay Kit (LABLEAD, Beijing, China) according to the manufacturer’s instructions. Concentrations were normalized to total protein content, expressed as μmol NAD^+^/NADH per milligram of protein. Relative NAD^+^/NADH levels were then analysed and compared with the control groups.

### Cell transfection

Cells were transfected with PMPCB, PMPCB-Myc and NDUFS8-Flag overexpression plasmids (all from Youbio Biotechnology, Hunan, China), PMPCB-targeting siRNA or miR-550a-5p mimics/inhibitors, along with mimics for miR-6804-5p, miR-4470, miR-597-3p and miR-4488 (all from Biomics Biotechnologies, Jiangsu, China), using Lipofectamine 3000 (Thermo Fisher Scientific). Cells were harvested 48 h post-transfection for downstream analysis (listed in Supplementary Tables 5, 6).

### Construction of reporter plasmids and dual luciferase reporter analysis

A DNA fragment containing the predicted miR-550a-5p binding site within the 3’ untranslated region (3’ UTR) of human PMPCB was amplified from genomic DNA by PCR. The PCR product was cloned into the pmirGLO dual-luciferase vector (RabbitBio, Chengdu, China) and verified by Sanger sequencing. To assess binding specificity, site-directed mutagenesis was performed on the putative miR-550a-5p binding site (5’-CAGGCAC-3’ → 5’-AGUUAGA-3’) in the PMPCB 3’ UTR. For luciferase assays, CD8^+^ T cells were co-transfected with either pmirGLO-PMPCB-WT or pmirGLO-PMPCB-Mut, and miR-550a-5p mimic or negative control mimic. Luciferase activity was measured 24 h post-transfection using the Dual-Luciferase Reporter Assay System (Promega, Madison, WI, USA). Firefly luciferase signals were normalized to Renilla luciferase activity for data analysis.

### RNA extraction and quantitative real-time polymerase chain reaction analysis

Total RNA was extracted from PMPs or CD8^+^ T cells using TRIzol reagent (Thermo Fisher Scientific). Reverse transcription was performed using The RevertAid First Strand cDNA Synthesis Kit (MCE, Monmouth Junction, NJ, USA) for mRNA analysis or the microRNA (miRNA) 1st Strand cDNA Synthesis Kit (Vazyme, Nanjing, China) for miRNA analysis. Quantitative PCR (qPCR) was carried out using SYBR Green Master Mix (MCE) on a real-time PCR system. Small nuclear RNA U6 (RNU6) and β-actin served as endogenous controls for miRNA and mRNA quantification, respectively. Relative gene expression was calculated using the 2^-^^ΔΔCt^ method. Primer sequences are provided in Supplementary Tables 7, 8.

### MicroRNA expression profiling

Total RNA was isolated from PMPs obtained from 5 CKD patients and 5 healthy controls using the TaKaRa RNAiso Plus reagent. The integrity and concentration of the extracted RNA were verified prior to library construction. Sequencing libraries were prepared and sequenced by BGI Genomics (Shenzhen, China) to generate miRNA expression profiles. Raw miRNA expression data were subjected to quantile normalization and log_2_ transformation. miRNAs with expression levels above the sample-specific median were classified as highly expressed. Differential expression analysis identified miRNAs with >1.2-fold upregulation or <0.833-fold downregulation for subsequent bioinformatic investigation.

### Western blot and immunoprecipitation

Mitochondria were isolated using a mitochondrial isolation kit (ACMEC, Shanghai, China). Proteins were extracted using RIPA lysis (Beyotime) buffer supplemented with protease and phosphatase inhibitors. For IP experiments, the supernatant was incubated with appropriate antibody-conjugated magnetic beads using a Dynabeads Co-Immunoprecipitation Kit (Thermo Fisher Scientific) following the manufacturer’s instructions. Protein expression levels were determined using anti-p21, anti-γ-H2A.X, anti-PMPCB, anti-NDUFS8, anti-CD63, anti-CD9, anti-TSG101, anti-β-actin and anti-COX IV. Then, the membranes were incubated with appropriate secondary antibodies for 1 h at 37°C and imaged by an Odyssey Infrared Imaging System (LI-COR Biosciences).

### Immunofluorescence

CD8^+^ T cells were fixed with 4% PFA, and blocked with 2% BSA. Cells were incubated overnight at 4°C with anti-p21, anti-γ-H2A.X or anti-PMPCB, followed by FITC-conjugated secondary antibody (37°C, 1 h). Nuclei were stained with DAPI (Beyotime). Visualization was performed using a Zeiss LSM800 NLO confocal microscopy (Carl Zeiss).

### Bioinformatics analysis

The human PBMC single-cell RNA sequencing dataset (GSE233315) was obtained from the NCBI Gene Expression Omnibus (GEO) database. Data processing and analysis were performed using the Seurat R package (version 5.3.0). Quality control filtering was applied as follows: cells with gene counts between 500 and 4,000 were retained, UMI counts >500 and <15,000 were included; cells exhibiting high ambient RNA contamination (log10(genes per UMI) ≤ 0.8) were excluded; cells with mitochondrial gene content exceeding 10% were removed.

### Statistical analysis

Statistical analyses were performed using GraphPad Prism 9.0 (GraphPad Software). Data are presented as mean ± standard deviation (SD). The number of independent biological replicates (*N*) is specified in the respective figure legends. Comparisons between two groups were analysed using paired or unpaired two-tailed Student’s *t* tests, as appropriate. Comparisons among three or more groups were analysed using one-way analysis of variance (ANOVA) followed by Tukey–Kramer post hoc tests. Associations between variables were assessed using Spearman’s rank correlation coefficient. Statistical significance was defined as *P* < 0.05 for all analyses.

## Results

### Peripheral blood CD8^+^ T cells develop premature senescence in patients with chronic kidney disease

**Fig. 1:**
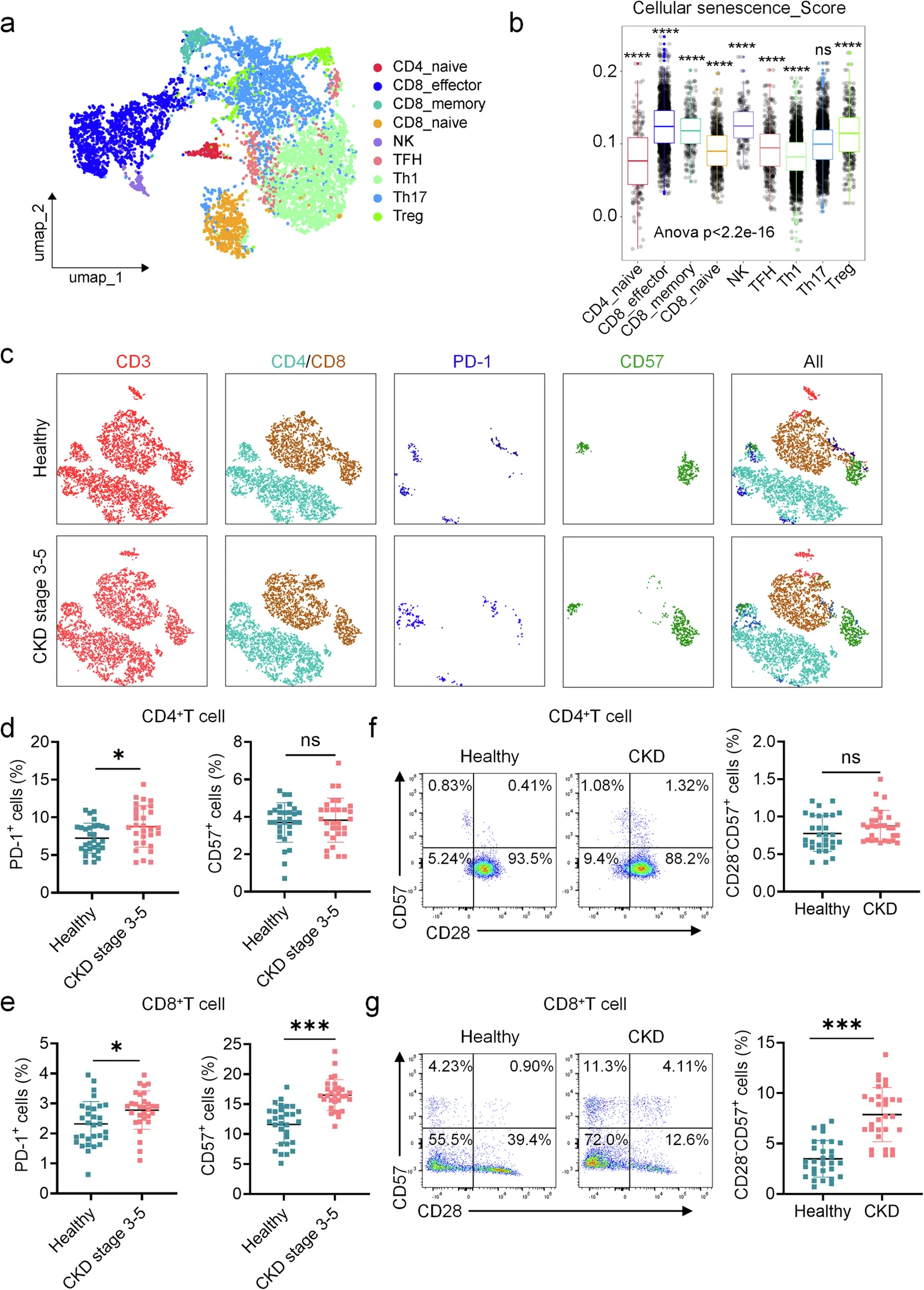
Peripheral blood CD8^+^ T cells develop premature senescence in patients with chronic kidney disease. **a** t-distributed stochastic neighbour embedding (t-SNE) projection of peripheral blood mononuclear cells from healthy controls and patients with end-stage renal disease, coloured by major cell clusters identified through small-cell RNA sequencing (scRNA-seq) analysis. Key T cell subsets and natural killer (NK) cells are indicated. TFH, T follicular helper cells. **b** Comparison of senescence scores across different T cell populations between the end-stage renal disease group and the healthy group. **c** t-SNE plot of T cells overlaid with expression of indicated cell-surface receptors determined by flow cytometry. **d**, **e** Quantitative analysis of the percentages of PD-1^+^ cells and CD57^+^ cells within CD4^+^ and CD8^+^ T cells between healthy controls and chronic kidney disease (CKD) stage 3–5 patients (*n* = 30). **f** **g** Representative flow cytometric analysis of the percentage of CD28^−^CD57^+^ cells within CD4^+^ and CD8^+^ T cells between healthy controls and CKD patients (*n* = 30). Data are presented as mean ± SD and were analysed using two-tailed, unpaired Student’s *t* test. ns no significance. *P < 0.05, ***P < 0.001.

Initially, we re-analysed published single-cell RNA sequencing (scRNA-seq) data from PBMCs obtained from healthy controls and patients with ESRD (GSE233315). Interestingly, we found that the markers of T cell exhaustion, PDCD1 and CTLA-4, were comparable between healthy controls and patients with ESRD, whereas Kyoto Encyclopedia of Genes and Genomes (KEGG) revealed a robust upregulation in cellular senescence score, particularly in CD8^+^ T effector cells (Fig. 1a, b and Supplementary Fig. 2a, b). Similarly, t-distributed stochastic neighbour embedding (t-SNE) analysis by flow cytometry identified a mild increase in PD-1 in CD4^+^ and CD8^+^ T cells from patients with CKD stage 3–5 compared with healthy controls (Fig. 1c–e and Supplementary Fig. 2c). Notably, CD57, a recognized marker used to identify terminally differentiated senescent T cells, and the proportion of CD57^+^ cells within the CD4^+^ T cell population showed no significant difference, whereas it was significantly higher within CD8^+^ T cells of patients with CKD stage 3–5 (Fig. 1c–e and Supplementary Fig. 2c). Furthermore, we found that CD57^+^CD8^+^ T cells were progressively developed along with advancing CKD and were most significant in CKD stage 5 patients (Supplementary Fig. 2d). Besides, CD28 is an age-related immune biomarker, and the loss of CD28 coupled with the presence of CD57 (CD28^−^CD57^+^) constitutes a key hallmark of immunosenescence. Flow cytometry also demonstrated that the proportion of CD28^–^CD57^+^ cells was significantly increased within the CD8^+^ T cell compartment of CKD patients, whereas there was no significant difference in their proportion within CD4^+^ T cells (Fig. 1f, g). Taken together, these data indicate that T cell immune incompetence develops in the pathophysiological context of CKD owing to multifaceted dysregulation, and that premature CD8^+^ T cell senescence potentially serves as a central contributor.

### CD8^+^ T cells exhibit upregulated senescence biomarkers accompanied by mitochondrial dysfunction in chronic kidney disease

**Fig. 2:**
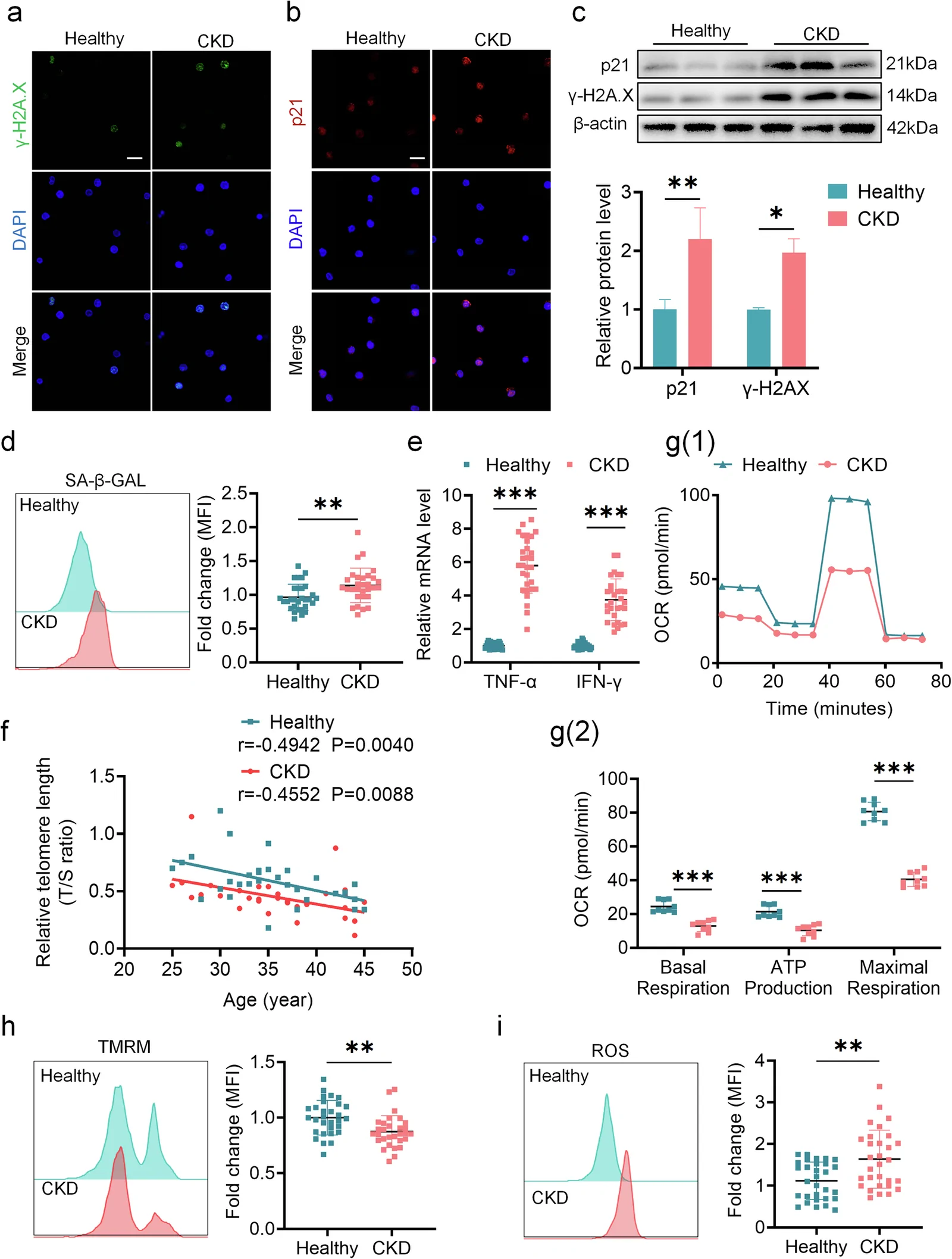
CD8^+^ T cells exhibit upregulated senescence biomarkers accompanied by mitochondrial dysfunction in chronic kidney disease. **a**, **b**, Representative immunofluorescence staining images showing γ-H2A.X and p21 expression in CD8^+^ T cells of healthy controls and patients with chronic kidney disease (CKD). Scale bar, 20 μm. **c** Western blot analysis of γ-H2A.X and p21 protein levels in CD8^+^ T cells of healthy controls and patients with CKD (*n* = 3). **d**, Flow cytometric analysis of senescence-associated β-galactosidase activity in CD8^+^ T cells of healthy controls and patients with CKD (*n* = 30). MFI, mean fluorescence intensity. **e** qPCR analysis of TNF and IFNγ expression in CD8^+^ T cells of healthy controls and patients with CKD (*n* = 30). **f** Correlation analysis between relative telomere length and age in CD8^+^ T cells of healthy controls and patients with CKD (*n* = 30). **g** Oxygen consumption rate (OCR) in CD8^+^ T cells of healthy controls and patients with CKD and quantitation of basal respiration, ATP-linked respiration, and maximal respiration (*n* = 9). **h**, **i** Flow cytometric analysis of tetramethylrhodamine methyl ester (TMRM) (**h**) and reactive oxygen species (ROS) (**i**) levels in CD8^+^ T cells of healthy controls and patients with CKD (*n* = 30). Data are presented as mean ± SD and were analysed using two-tailed, unpaired Student’s *t* test and Pearson’s correlation analysis (**f**). *P < 0.05, **P < 0.01, ***P < 0.001.

Cellular senescence is defined as the irreversible loss of replicative potential caused by persistent DNA damage, accompanied by increased β-galactosidase activity, cell-cycle arrest and telomere shortening^15^. Indeed, we observed significantly elevated expression of the DNA damage marker γ-H2A.X and the cyclin-dependent kinase inhibitor p21 in CD8^+^ T cells from CKD patients compared with healthy controls (Fig. 2a–c and Supplementary Fig. 3a, b), as well as significantly increased SA-β-Gal activity and expression of the senescent proinflammatory cytokines tumour necrosis factor (TNF) and interferon gamma (IFNγ) (Fig. 2d, e). Meanwhile, relative telomere length analysis revealed progressive age-dependent telomere shortening in CD8^+^ T cells in both groups, with significantly shorter telomeres observed in CKD patients (Fig. 2f). Furthermore, senescence-associated mitochondrial dysfunction is considered an essential component of the senescent phenotype^26^. Accordingly, we detected a decrease in OCR, maximal respiratory capacity and ATP production (Fig. 2g). Mitochondrial dysfunction was further confirmed by the loss of mitochondrial membrane potential (Fig. 2h). Mitochondria are sensitive to oxidative stress, which is the most prominent characteristic of the CKD milieu, and we also observed ROS overproduction in CD8^+^ T cells of patients with CKD (Fig. 2i). Collectively, these findings demonstrate that CD8^+^ T cells from CKD patients exhibit a comprehensive senescent phenotype characterized by well-established senescence markers and severe mitochondrial dysfunction.

### Platelets from patients with chronic kidney disease induce premature CD8^+^ T cell senescence by mediating mitochondrial dysfunction

**Fig. 3:**
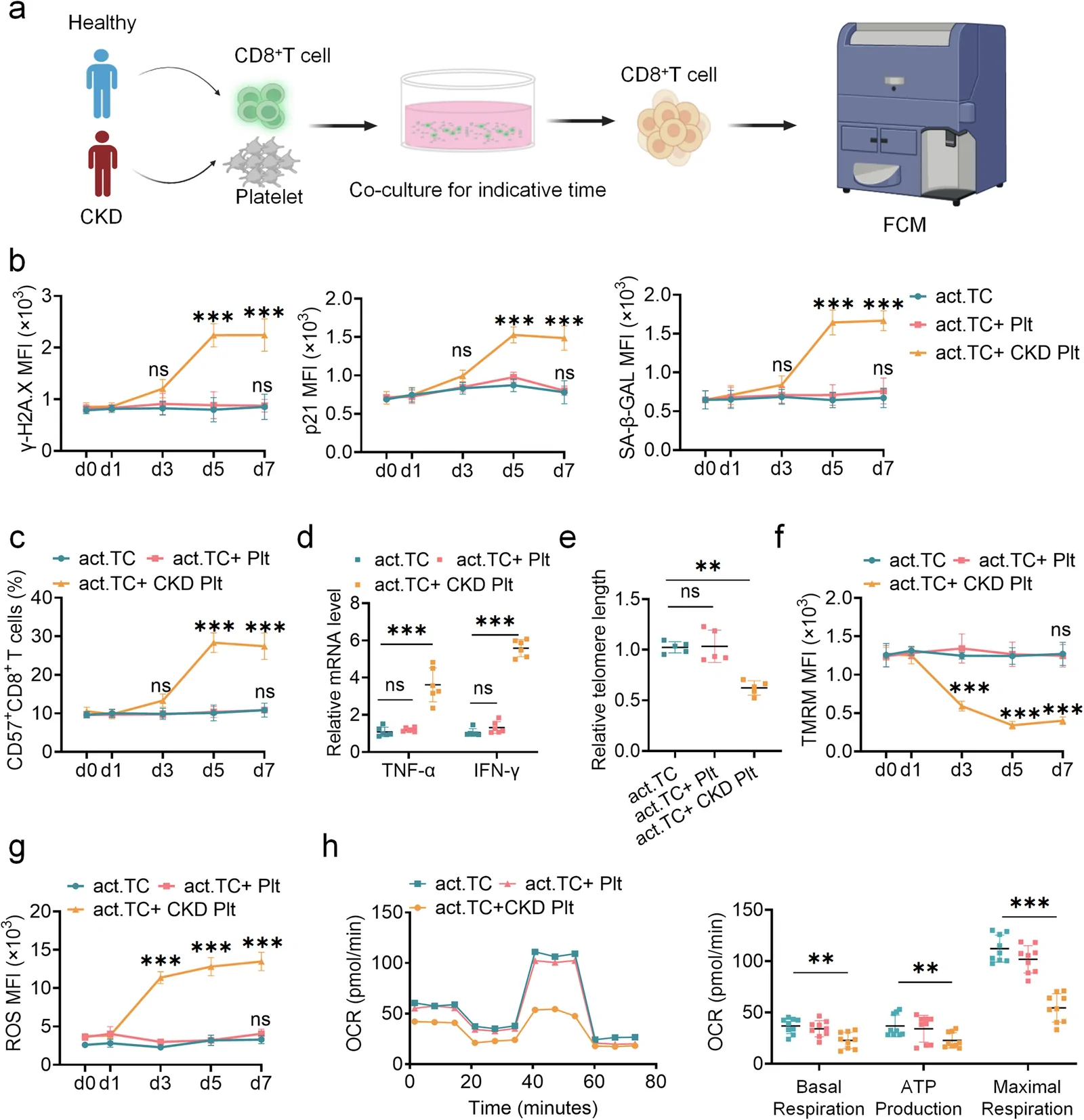
Platelets from patients with chronic kidney disease induce premature CD8^+^ T cell senescence by mediating mitochondrial dysfunction. **a** Schematic diagram illustrating the co-culture system of platelets and CD8^+^ T cells. CD8^+^ T cell activation was induced using CD3/CD28 antibodies. **b** Flow cytometric (FCM) analysis of γ-H2A.X and p21 expression levels, and senescence-associated β-galactosidase (SA-β-Gal) activity in CD8^+^ T cells co-cultured with platelets (Plt) from healthy controls or patients with chronic kidney disease (CKD) (*n* = 3). MFI, mean fluorescence intensity. **c** Flow cytometric analysis of the proportion of CD57^+^ cells in CD8^+^ T cells co-cultured with platelets from healthy controls or patients with CKD (*n* = 3). **d** Quantitative PCR analysis of TNF and IFNγ expression in CD8^+^ T cells from different groups (*n* = 6). **e** Relative telomere length in CD8^+^ T cells from different groups (*n* = 6). **f**, **g** Flow cytometric analysis of tetramethylrhodamine methyl ester (TMRM) (**f**) and reactive oxygen species (ROS) (**g**) levels in CD8^+^ T cells across different groups (*n* = 3 for each analysis). **h** Oxygen consumption rate (OCR) in CD8^+^ T cells from different groups, with quantitation of basal respiration, ATP production, and maximal respiration (*n* = 9). act. TC, activated CD8^+^ T cells. Data are presented as mean ± SD and were analysed by one-way analysis of variance. ns no significance. **P < 0.01, ***P < 0.001.

We next sought to identify the role of platelets from patients with CKD in premature CD8^+^ T cell senescence. We first established a co-culture system in which platelets derived from healthy donors or patients with CKD, respectively, were co-cultured with CD8^+^ T cells derived from healthy donors (Fig. 3a). As shown, CD8^+^ T cells cultured alone or co-cultured with platelets derived from healthy individuals did not show a cellular senescence phenotype over time (Fig. 3b–e). However, CD8^+^ T cells co-cultured with platelets derived from patients with CKD exhibited a dramatic acceleration of premature CD8^+^ T cell senescence by day 5, as demonstrated by the upregulation of γ-H2A.X, p21, SA-β-Gal and CD57 (Fig. 3b, c). Besides, upregulation of senescent proinflammatory cytokines and shortened telomeres were also detected in CD8^+^ T cells after co-culture with platelets from patients with CKD (Fig. 3d, e). Surprisingly, the mitochondrial membrane potential decreased, and impaired mitochondrial respiratory function and ROS overproduction were observed in CD8^+^ T cells at day 3 after co-culture with platelets from patients with CKD, preceding the appearance of cellular senescence (Fig. 3f–h). In contrast, pretreatment with N-acetylcysteine (NAC), an antioxidant agent, effectively prevented mitochondrial dysfunction and cellular senescence in CD8^+^ T cells induced by platelets from patients with CKD (Supplementary Fig. 4), indicating that platelets from patients with CKD might be involved in the initiation of premature CD8^+^ T cell senescence through mediating mitochondrial dysfunction.

### Platelet-derived microparticles from patients with chronic kidney disease mediate mitochondrial dysfunction and cellular senescence in CD8^+^ T cells

**Fig. 4:**
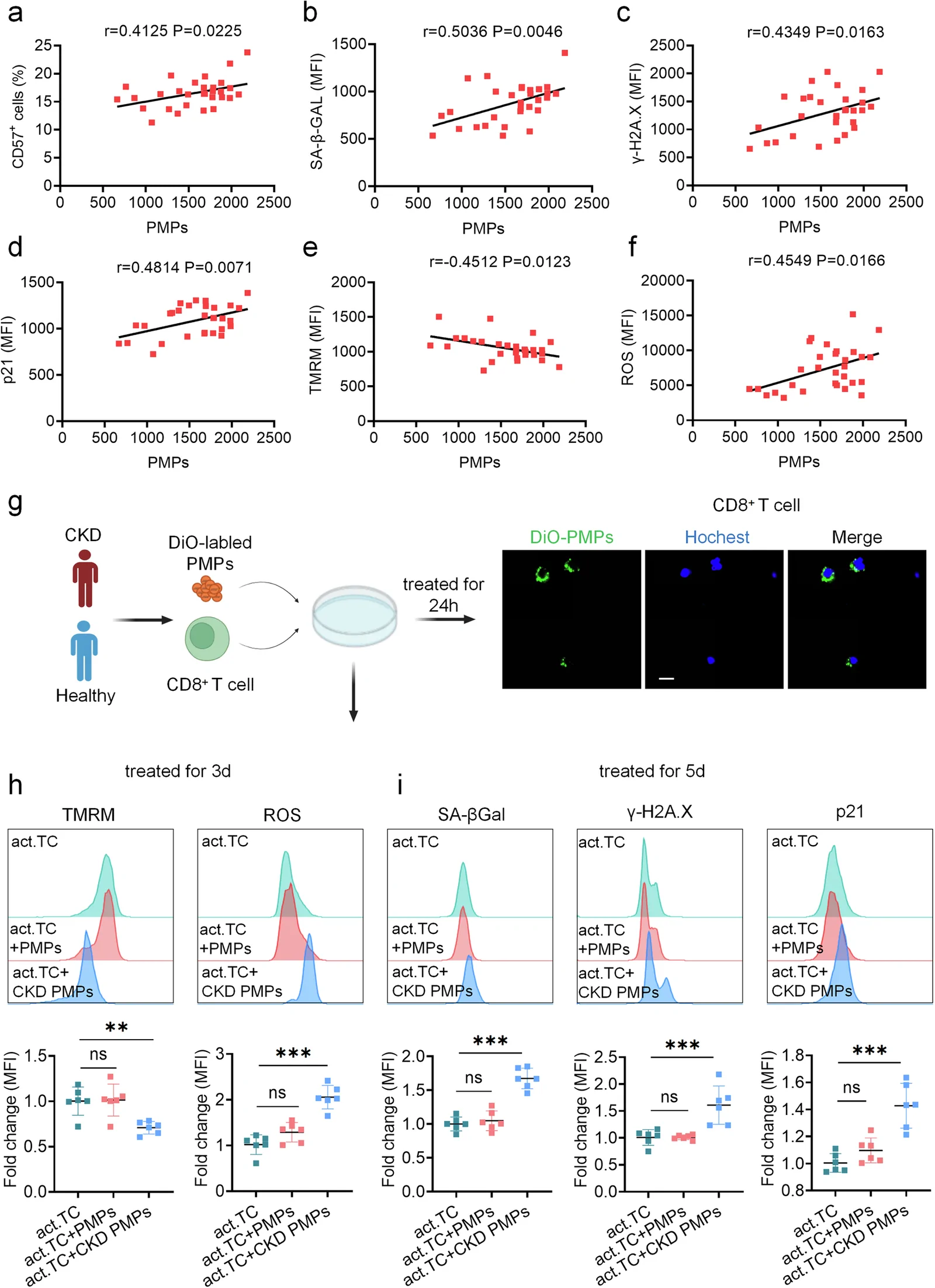
Platelets-derived microparticles released from patients with chronic kidney disease mediate mitochondrial dysfunction and cellular senescence in CD8^+^ T cells. **a–f** Correlation analysis between the levels of platelets from microparticles (PMPs) in peripheral blood and the proportion of CD57^+^ cells (**a**), senescence-associated β-galactosidase (SA-β-Gal) activity (**b**), γ-H2A.X level (**c**), p21 expression (**d**), tetramethylrhodamine methyl ester (TMRM) levels (**e**) and reactive oxygen species (ROS) levels (**f**) in CD8^+^ T cells from patients with CKD (*n* = 30). MFI, mean fluorescence intensity. **g** Schematic diagram and representative immunofluorescence images illustrating the internalization of PMPs by CD8^+^ T cells. PMPs were labelled with DiO (green), and nuclei of CD8^+^ T cells were stained with Hoechst (blue). Scale bar, 20 μm. **h**, **i** Mitochondrial function was assessed in cells collected on day 3, and senescence markers were analysed on day 5. Flow cytometric analysis of TMRM levels, and ROS levels (**h**), SA-β-Gal activity, γ-H2A.X level, p21 expression (**i**) in CD8^+^ T cells treated with PMPs from healthy controls or patients with CKD (*n* = 6). act. TC, activated CD8^+^ T cells. Data are presented as mean ± SD and were analysed by one-way analysis of variance or Pearson’s correlation analysis (**a–f**). **P < 0.01, ***P < 0.001.

Previously, we have shown that CKD promotes platelet hyperreactivity, inducing the large-scale release of PMPs, which have been reported to mediate mitochondrial dysfunction^19,27^. Consistently, remarkably increased platelet activity including P-selectin, activated GPIIb/IIIa expression and PMP release were verified in patients with CKD (Supplementary Fig. 5a, b). Notably, PMP release in the peripheral blood of patients with CKD correlated positively with cellular senescence biomarkers and ROS levels in CD8^+^ T cells, whereas it correlated negatively with mitochondrial membrane potential, hinting that PMP release may facilitate mitochondrial dysfunction and cellular senescence of CD8^+^ T cells in the CKD milieu (Fig. 4a–f). To investigate whether PMPs directly regulate CD8^+^ T cell senescence, we isolated PMPs and characterized their biological and morphological properties (Supplementary Fig. 5c–f). CD8^+^ T cells were then treated with PMPs ex vivo and DiO-labelled PMPs were successfully internalized by CD8^+^ T cells (Fig. 4g). Consistently, after tail-vein injection of PMPs, DiO-positive signals were detected in mouse peripheral blood CD8^+^ T cells (Supplementary Fig. 5g). Furthermore, PMPs derived from patients with CKD significantly induced mitochondrial dysfunction and cellular senescence in CD8^+^ T cells, whereas NAC dramatically relieved these effects (Fig. 4h, i and Supplementary Fig. 6). Particularly, CKD-derived PMPs neither significantly affected the proliferation of CD8^+^ T cells nor aggravated the senescence of CD57^+^CD8^+^ T cells but mainly promoted the transition of CD57^−^CD8^+^ T cells towards a CD57^+^ senescent phenotype (Supplementary Fig. 7). Hence, these results demonstrate that a direct action of PMPs on premature CD8^+^ T cells senescence by inducing mitochondrial dysfunction.

### Platelet-derived microparticles promote premature CD8^+^ T cell senescence in an miR-550a-5p-dependent manner

**Fig. 5:**
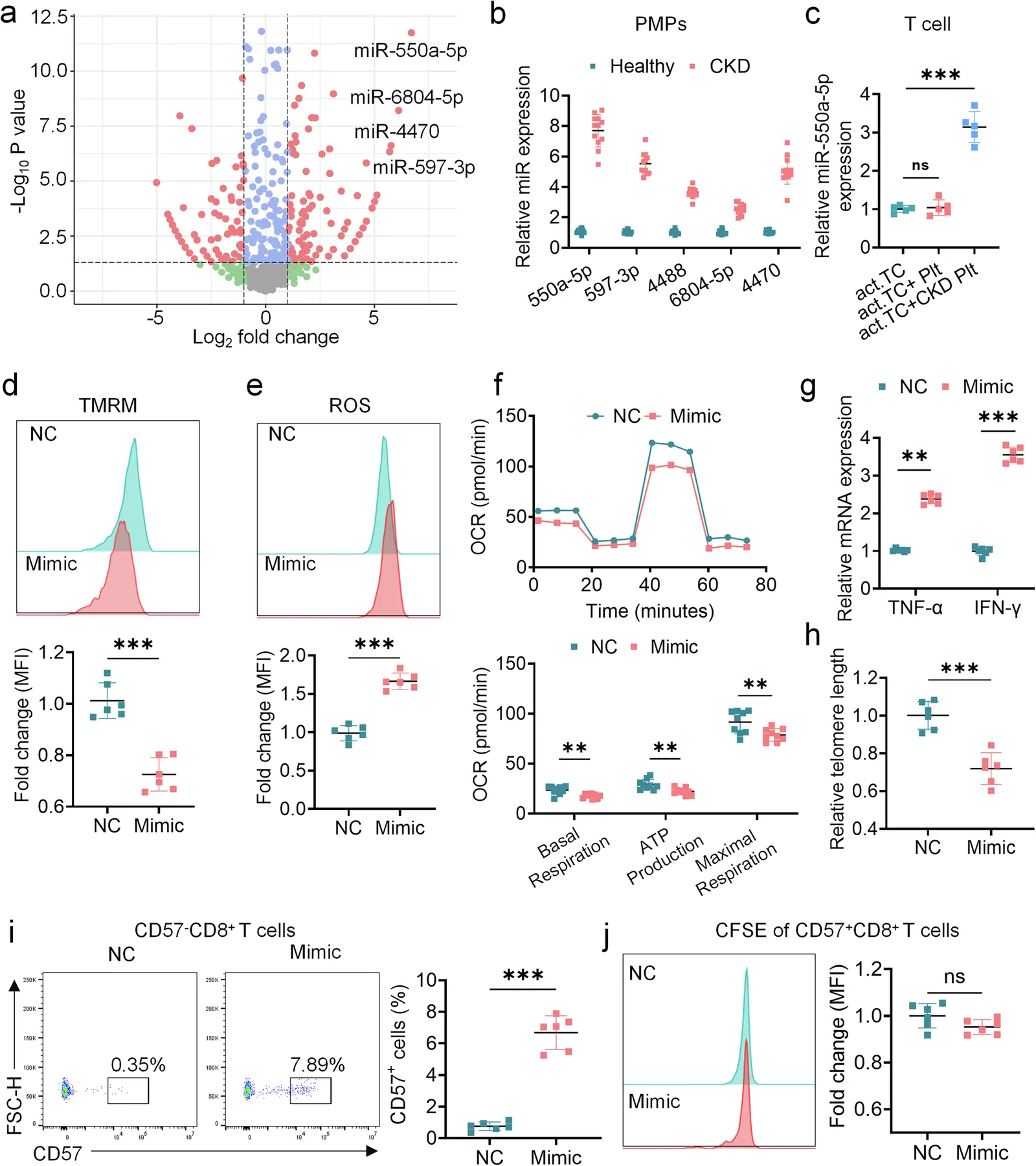
Platelet-derived microparticles promote premature CD8^+^ T cell senescence in an miR-550a-5p-dependent manner. **a** Volcano plot illustrating differentially expressed microRNAs (miRNAs) between healthy controls and patients with chronic kidney disease (CKD), with thresholds set at |log₂(fold change)| > 0.263 (equivalent to >1.2 or <0.833-fold change) and *P* < 0.05. **b** Quantitative PCR (qPCR) analysis of the expression of selected miRNAs in platelet-derived microparticles (PMPs) of healthy controls and patients with CKD (*n* = 12). **c** qPCR analysis of miR-550a-5p expression in CD8^+^ T cells co-cultured with platelets (Plt) from healthy controls and patients with CKD (*n* = 6). **d**, **e** Flow cytometric analysis of tetramethylrhodamine methyl ester (TMRM) levels (**d**), and reactive oxygen species (ROS) levels (**e**) in CD8^+^ T cells treated with or without miR-550a-5p mimic (*n* = 6). MFI, mean fluorescence intensity. **f** Oxygen consumption rate (OCR) in CD8^+^ T cells treated with or without miR-550a-5p mimic and quantitation of basal respiration, ATP production and maximal respiration (*n* = 9). NC, negative control. **g**, **h** qPCR analysis of TNF and IFNγ expression (**g**) and relative telomere length (**h**) in CD8^+^ T cells treated with or without miR-550a-5p mimic (*n* = 6). **i** Flow cytometric analysis of the proportion of CD57^+^ cells in CD57^−^CD8^+^ T cells treated with miR^−^550a-5p mimic or control mimic (*n* = 6). FSC-H, forward scatter height. **j** Flow cytometric analysis of carboxyfluorescein diacetate succinimidyl ester (CFSE) in CD57^+^CD8^+^ T cells treated with miR-550a-5p mimic or control mimic (*n* = 6). act. TC, activated CD8^+^ T cells. Data are presented as mean ± SD and were analysed using two-tailed, unpaired Student’s *t* test or one-way analysis of variance (**c**). ns no significance. **P < 0.01, ***P < 0.001.

PMPs are enriched in platelet microRNAs, which play an important role in regulating mitochondrial dysfunction and cellular senescence^28^. PMPs isolated from healthy controls and patients with CKD showed profound expression differences, with 109 upregulated and 57 downregulated miRNAs in CKD-derived PMPs (Fig. 5a). Among them, miR-550a-5p, miR-6804-5p, miR-4470, miR-597-3p and miR-4488 were the top five, with miR-550a-5p being the most remarkably upregulated (Fig. 5a, b). CD8^+^ T cells transfected with corresponding miRNA mimics revealed that only miR-550a-5p significantly increased SA-β-Gal positivity, γ-H2A.X expression and p21 expression (Supplementary Fig. 8a–e). Unexpectedly, Starbase, TargetScan and miRDB databases revealed that miR-550a-5p was not expressed in mice (data not shown). Notably, the miR-550a-5p content in CD8^+^ T cells was much lower than in PMPs, whereas the miR-550a-5p content in CD8^+^ T cells of patients with CKD was higher than in healthy controls, suggesting that miR-550a-5p in CKD-derived PMPs was transferred to CD8^+^ T cells (Supplementary Fig. 8f), which were also observed in the co-cultured system (Fig. 5c). In MEG-01 cells, only CKD serum (not PBMCs) significantly elevated miR-550a-5p expression (Supplementary Fig. 8 g), implying that soluble factors drive its upregulation. Subsequently, we validated that miR-550a-5p overexpression in CD8^+^ T cells elevated other senescent phenotypes and mitochondrial dysfunction (Fig. 5d–h and Supplementary Fig. 8h), mainly by promoting the CD57^−^ to CD57^+^ conversion (Fig. 5i, j). Conversely, treating CD8^+^ T cells with miR-550a-5p inhibitor rescued these effects induced by platelets from patients with CKD under the co-cultured system (Supplementary Fig. 9). Collectively, these results demonstrate that PMPs from patients with CKD induce CD8^+^ T cell senescence via a miR-550a-5p-mediating mitochondrial dysfunction axis.

### MiR-550a-5p induces mitochondrial dysfunction and cellular senescence in CD8^+^ T cells via targeting PMPCB

**Fig. 6:**
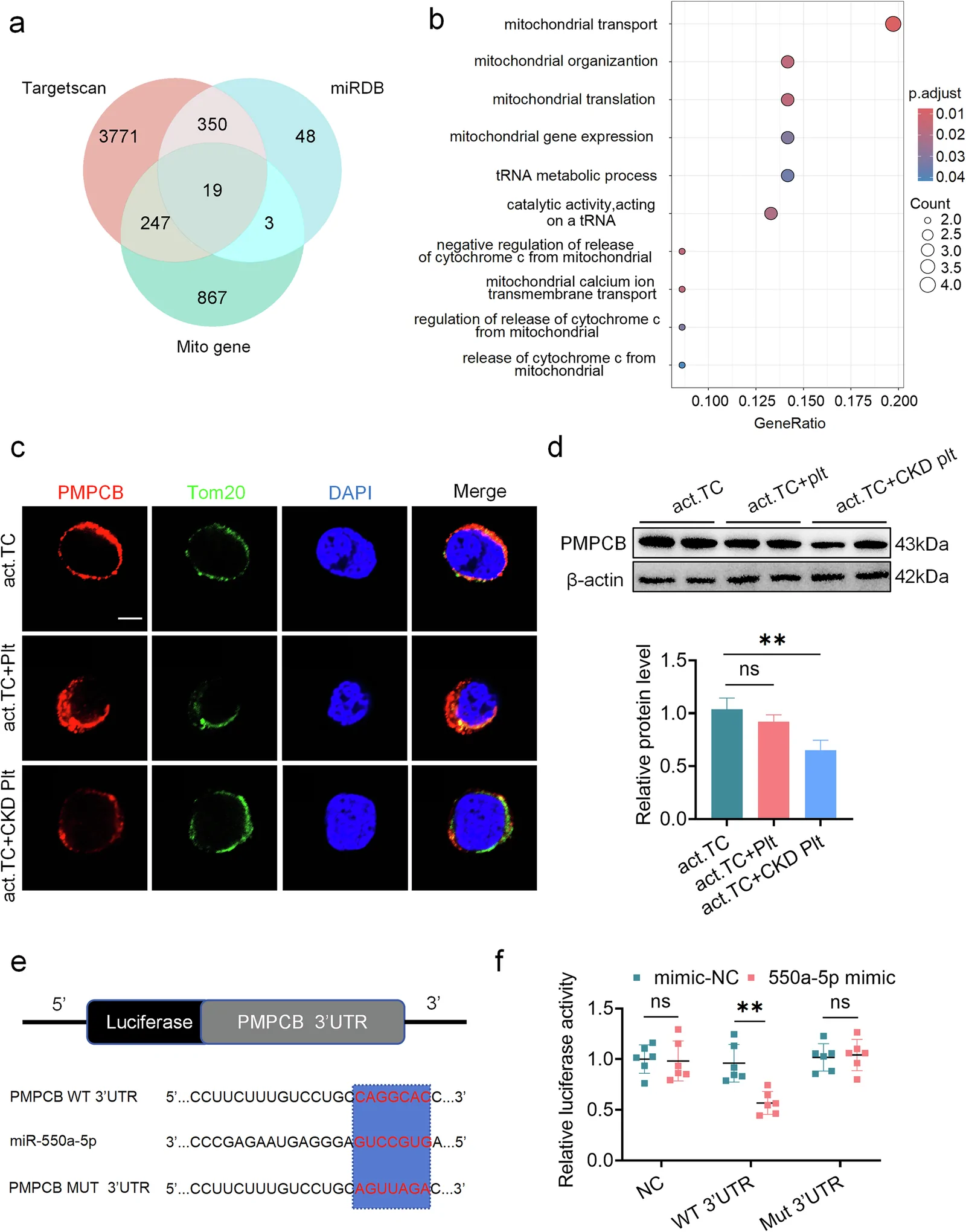
MiR-550a-5p regulates PMPCB expression via directly binding to its 3’untranslated region. **a** Venn diagram showing the overlap between predicted target genes of miR-550a-5p from TargetScan and miRDB databases and human mitochondrial genes sourced from the MitoCarta database. **b** Gene ontology enrichment analysis of the predicted mitochondrial target genes. tRNA, transfer RNA. **c** Representative immunofluorescence staining images showing PMPCB expression (red) in CD8^+^ T cells co-cultured with platelets (Plt) from healthy controls or patients with chronic kidney disease (CKD). Mitochondria were labelled with Tom20 (green). Scale bar, 10 μm. **d** Western blot analysis of PMPCB protein levels in CD8^+^ T cells co-cultured with platelets from healthy controls or patients with CKD (*n* = 3). **e** Schematic illustration of the wild-type (WT) and mutant (Mut) putative miR-550a-5p binding sites within the 3′ untranslated region (UTR) of the PMPCB gene cloned into a luciferase reporter vector. **f** Relative luciferase activity in CD8^+^ T cells co-transfected with either the WT or Mut PMPCB 3′ UTR reporter plasmid and miR-550a-5p mimic or negative control (NC) mitochondrial RNA. Luciferase activity was normalized to Renilla luciferase activity (*n* = 6). act. TC, activated CD8^+^ T cells. Data are presented as mean ± SD and were analysed using two-tailed, unpaired Student’s *t* test or one-way analysis of variance (**d**). ns no significance. **P < 0.01.

To investigate the underlying mechanisms by which miR-550a-5p regulates CD8^+^ T cells mitochondrial dysfunction-induced cellular senescence, bioinformatic analysis (TargetScan, miRDB, MitoCarta 3.0) predicted 19 potential mitochondrial target genes (Fig. 6a and Supplementary Table 9). Gene Ontology (GO) enrichment analysis showed significant enrichment in mitochondrial protein transport (Fig. 6b), suggesting that miR-550a-5p may disrupt mitochondrial transport processes. Given that miRNAs negatively regulate gene expression post-transcriptionally^29^, we observed that PMPCB expression was significantly decreased at both mRNA and protein levels in CD8^+^ T cells of patients with CKD (Supplementary Fig. 10a–c). Consistently, miR-550a-5p mimic transfection selectively reduced PMPCB levels (Supplementary Fig. 10d). PMPCB is the catalytic subunit of mitochondrial processing peptidase, which promotes the maturation of precursor proteins transported to mitochondria^30^. Besides, co-culture with platelets from patients with CKD also reduced PMPCB expression in CD8^+^ T cells (Fig. 6c, d and Supplementary Fig. 10e), similar to miR-550a-5p overexpression (Supplementary Fig. 10f, g). Furthermore, bioinformatic analysis predicted perfect complementary sequences between miR-550a-5p and the PMPCB 3’-untranslated region (UTR) (Fig. 6e). Dual-luciferase reporter assays confirmed that miR-550a-5p overexpression decreased luciferase activity of the wild-type but not the mutant (Fig. 6f). Interestingly, interrogation of sequences in the 3’UTR of mouse PMPCB gene also identified a miR-550a-5p binding site (Supplementary Fig. 10h). Moreover, mice treated with miR-550a-5p agomir exhibited cellular senescence, mitochondrial dysfunction and PMPCB downregulation in CD8^+^ T cells (Supplementary Fig. 10i and Supplementary Fig. 11). Collectively, these results demonstrate that miR-550a-5p could directly bind and downregulate PMPCB expression.

**Fig. 7:**
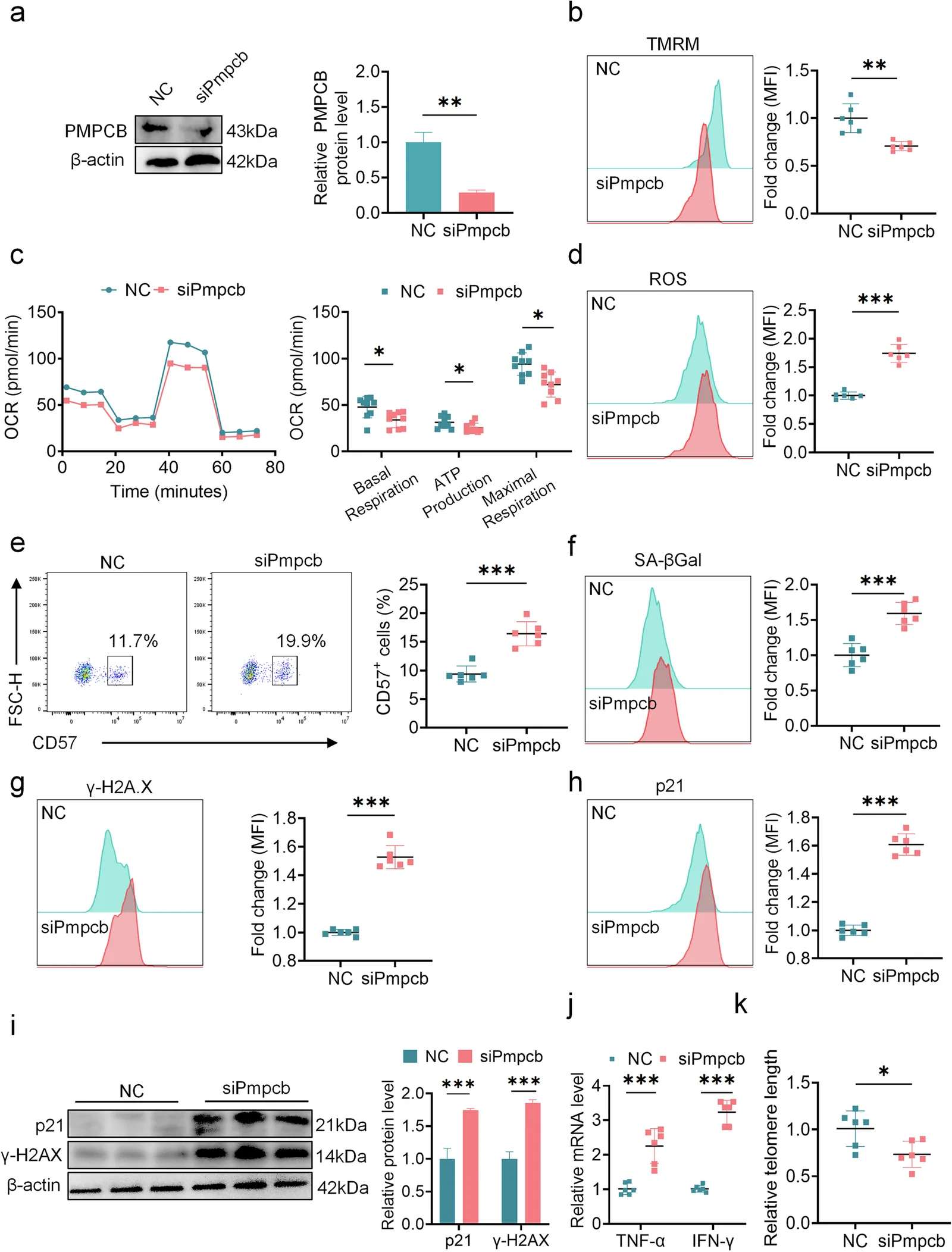
Knockdown of PMPCB induces mitochondrial dysfunction and cellular senescence in CD8^+^ T cells. **a** Western blot analysis of PMPCB protein levels in CD8e-k T cells treated with PMPCB-specific small interfering RNA (siRNA) or control siRNA (*n* = 3). NC, negative control. **b** Flow cytometric analysis of tetramethylrhodamine methyl ester (TMRM) levels in CD8^+^ T cells treated with PMPCB-specific siRNA or control siRNA (*n* = 6). MFI, mean fluorescence intensity. **c** Oxygen consumption rate (OCR) in CD8^+^ T cells treated with PMPCB-specific siRNA or control siRNA, with quantitation of basal respiration, ATP production and maximal respiration (*n* = 9). **d–h** Flow cytometric analysis of reactive oxygen species (ROS) levels (**d**), proportion of CD57^+^ cells (**e**), senescence-associated β-galactosidase (SA-β-Gal) activity (**f**) γ-H2A.X level (**g**) and p21 expression (**h**) in CD8^+^ T cells treated with PMPCB-specific siRNA or control siRNA (*n* = 6). FSC-H, forward scatter height. **i** Western blot analysis of γ-H2A.X and p21 protein levels in CD8^+^ T cells treated with PMPCB-specific siRNA or control siRNA (*n* = 3). **j**, **k** Quantitative PCR analysis of TNF and IFNγ expression (**j**) and relative telomere length (**k**) in CD8^+^ T cells treated with PMPCB-specific siRNA or control siRNA (*n* = 6). Data are presented as mean ± SD and were analysed using a two-tailed, unpaired Student’s *t* test. ns no significance. *P < 0.05, **P < 0.01, ***P < 0.001.

To determine the role of PMPCB in CD8^+^ T cells, we first transfected siRNA against PMPCB in CD8^+^ T cells ex vivo (Fig. 7a). As expected, the absence of PMPCB induced decreased mitochondrial membrane potential and impaired mitochondrial respiratory function, but increased ROS levels (Fig. 7b–d). Consistently, it also upregulated cellular senescence biomarkers, pro-inflammatory cytokines, and shortened telomeres, indicating its important role in maintaining mitochondrial homeostasis (Fig. 7e–k and Supplementary Fig. 12). Conversely, PMPCB overexpression alleviated miR-550a-5p-induced mitochondrial dysfunction, ultimately mitigating cellular senescence in CD8e-k T cells (Supplementary Fig. 13). Taken together, these data underscore a critical role of miR-550a-5p in mediating premature CD8^+^ T cell senescence by targeting PMPCB, which is essential for the maintenance of mitochondrial homeostasis.

### Nicotinamide mononucleotide counteracts platelets from patients with chronic kidney disease-induced and NDUFS8-mediated cellular senescence in CD8^+^ T cells

**Fig. 8:**
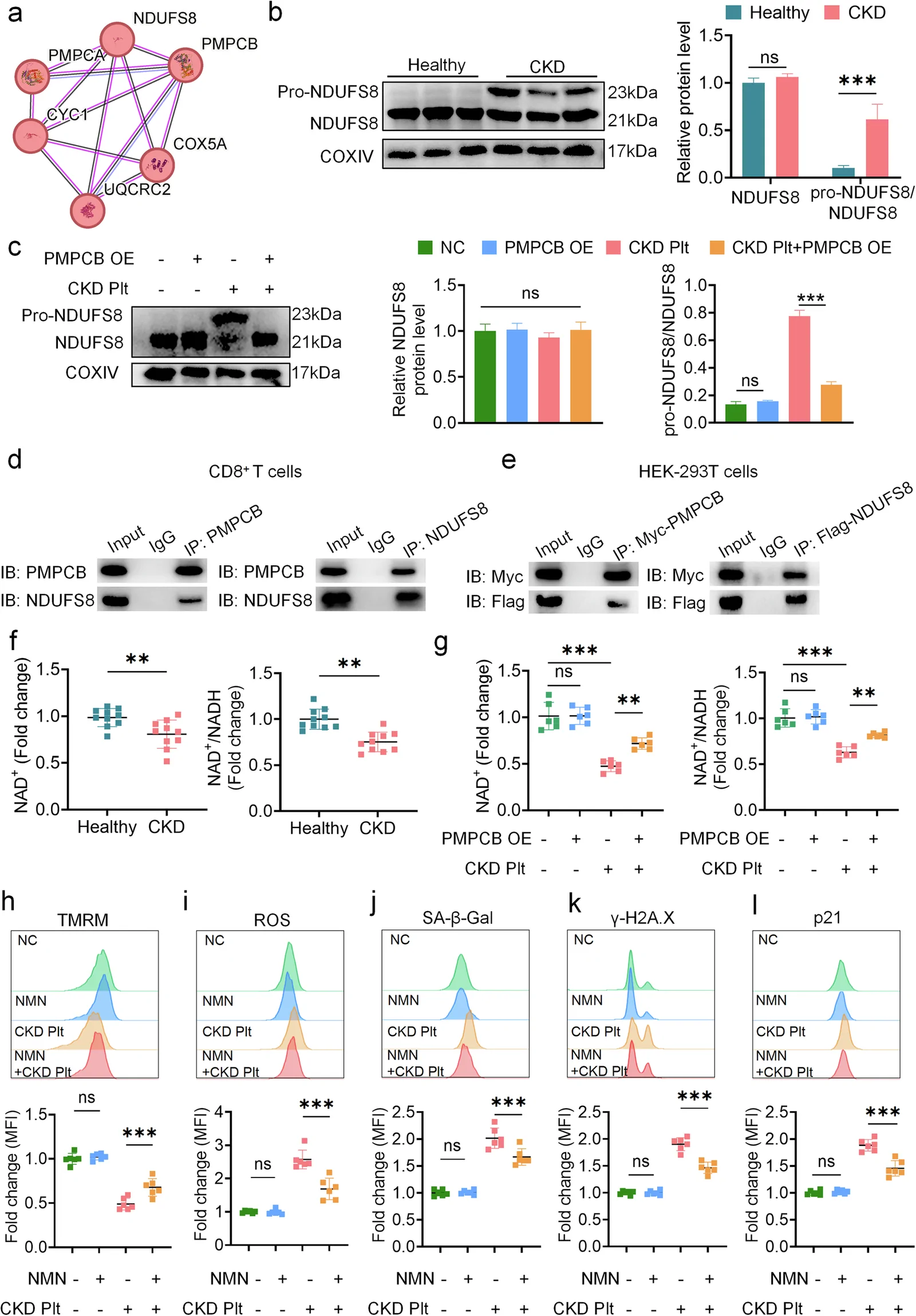
Nicotinamide mononucleotide counteracts platelets from patients with chronic kidney disease-induced and NDUFS8-mediated cellular senescence in CD8^+^ T cells. **a** Prediction of PMPCB-interacting proteins using the STRING database platform. **b** Western blot analysis of NDUFS8 expression in CD8^+^ T cells of healthy controls and patients with chronic kidney disease (CKD) (*n* = 3). **c** Western blot analysis of NDUFS8 expression in CD8^+^ T cells transfected with or without PMPCB overexpression (OE) plasmids under a co-cultured system (*n* = 3). Plt, platelets. **d** Reciprocal co-immunoprecipitation analysis of interaction between PMPCB and NDUFS8 in human CD8^+^ T cells. **e** HEK293T cells were co-transfected with PMPCB-Myc and NDUFS8-Flag plasmids. Reciprocal co-immunoprecipitation analysis of interaction between PMPCB and NDUFS8. NC, negative control. **f** Measurement of NAD^+^ content and NAD^+^/NADH ratio in CD8^+^ T cells of healthy controls and patients with CKD (*n* = 10). **g** Measurement of NAD^+^ content and NAD^+^/NADH ratio in CD8^+^ T cells transfected with or without PMPCB overexpression plasmids under a co-cultured system (*n* = 6). **h–l** Flow cytometric analysis of tetramethylrhodamine methyl ester (TMRM) levels (**h**), reactive oxygen species (ROS) levels (**i**), senescence-associated β-galactosidase (SA-β-Gal) activity (**j**), γ-H2A.X level (**k**) and p21 expression (**l**) in CD8^+^ T cells pretreated with or without 100 µM nicotinamide mononucleotide (NMN) under a co-cultured system (*n* = 6). MFI, mean fluorescence intensity. Data are presented as mean ± SD and were analysed using the two-tailed, unpaired Student’s *t* test (**b**, **f**) or one-way analysis of variance. ns no significance. **P < 0.01 and ***P < 0.001.

To further elucidate PMPCB-mediated CD8^+^ T cell senescence mechanisms, we identified six PMPCB-interacting proteins using a protein–protein interaction analysis (Fig. 8a). Notably, NDUFS8 is a subunit of monitoring NADH dehydrogenase in mitochondrial complex I, which would be cleaved by mitochondrial processing peptidase^31^. We found no significant difference in NDUFS8 expression in CD8^+^ T cells between patients with CKD and healthy controls, nor upon co-culture with CKD patient platelets or miR-550a-5p mimic/agomir treatment (Fig. 8b, c and Supplementary Fig. 14a–d). Interestingly, we detected the precursor and mature forms of NDUFS8 in CD8^+^ T cells of patients with CKD, whereas only the mature form of NDUFS8 existed in CD8^+^ T cells of healthy controls (Fig. 8b). Moreover, co-culture with platelets of patients with CKD or treatment with miR-550a-5p mimic in human and miR-550a-5p agomir in mouse CD8^+^ T cells induced cleavage failure of NDUFS8, whereas overexpression of PMPCB significantly alleviated these effects (Fig. 8c and Supplementary Fig. 14c, d). Co-immunoprecipitation experiments verified an interaction between PMPCB and NDUFS8 in CD8^+^ T cells (Fig. 8d, e), indicating that PMPCB regulates NDUFS8 cleavage. NDUFS8 suppression will impair electron transfer from NADH to ubiquinone, thereby decreasing NAD^+^/NADH ratio, which has been reported to drive cells into senescence^32,33^. Indeed, patients with CKD exhibited decreased NAD^+^ levels and NAD^+^/NADH ratios in CD8^+^ T cells (Fig. 8f). Similar reductions were observed upon co-culture with CKD patient platelets or miR-550a-5p mimic treatment, whereas PMPCB overexpression partially ameliorated this effect (Fig. 8g and Supplementary Fig. 14e). Therefore, we treated CD8^+^ T cells with NMN, an intermediate in NAD^+^ biosynthesis^34^. NMN treatment effectively attenuated mitochondrial dysfunction in CD8^+^ T cells, thereby preventing platelets from patients with CKD-induced cellular senescence (Fig. 8h–l and Supplementary Fig. 14f–i). Moreover, we performed an intraperitoneal injection of NMN (500 mg/kg per mouse) once every 3 days in miR-550a-5p agomir-treated mice for 4 weeks. Consistently, NMN treatment effectively alleviated mitochondrial dysfunction and cellular senescence in CD8^+^ T cells of mice (Supplementary Fig. 15). Above all, these findings indicate that NMN may serve as a promising therapeutic drug for alleviating platelets from patients with CKD-mediated mitochondrial dysfunction and cellular senescence.

## Discussion

Infection is a leading cause of morbidity and mortality in patients with CKD^3^. T cell activation significantly influences an individual’s immune function and susceptibility to infection^6^. Although disturbances in T cell homeostasis in CKD have been extensively studied, significant knowledge gaps remain regarding the underlying mechanisms of T cell dysfunction^10^. In this study, we identify that premature CD8^+^ T cell senescence is a key feature of altered T cell homeostasis in patients with CKD. We further demonstrate that platelets accelerate this senescence process by transferring PMPs containing miR-550a-5p into CD8^+^ T cells, thereby downregulating PMPCB expression and inhibiting the NDUFS8/NAD^+^ axis. Conversely, inhibiting miR-550a-5p function or overexpressing PMPCB ameliorated platelet-induced premature CD8^+^ T cell senescence in CKD.

Patients with CKD are more prone to develop premature age-related diseases^11^. Immunosenescence contributes to the development of infections, cancers and autoimmune diseases, as well as accelerating the progression of fatal diseases, including cardiovascular and metabolic disorders, non-alcoholic fatty liver disease and neurodegenerative diseases^35,36^. However, the characteristics and molecular mechanisms underlying immune-cell senescence in CKD remain incompletely understood. In this study, integrated bioinformatic analysis and clinical sample validation revealed that CD8^+^ T cells exhibit more pronounced senescent change than CD4^+^ T cells, which is consistent with previous reports^9,37^. Currently, the cause of T cell senescence in CKD is attributed to the chronic inflammation resulting from uraemia or maintenance dialysis^11^. In our study, we enrolled patients who were not on dialysis, and observed that CD8^+^ T cell senescence progressively developed along with advancing CKD. Besides, given the close association between CD8^+^ T cell senescence and CMV infection in patients with CKD, we excluded CMV-seropositive individuals at enrollment^11^. Interestingly, co-cultured with platelets from patients with CKD represent anothors than can induce CD8^+^ T cell senescence, with cells derived from healthy controls. Although the role of platelets in haemostasis is well known, they are also considered a cellular component of the innate immune system^17^. Based on our findings, we discovered that, in addition to uraemia, platelets can serve as immune regulatory cells to mediate CD8^+^ T cell senescence.

As already known, platelets can directly interact with immune cells via specific receptors, such as CD41, CD42 and CD154, but our study excluded this effect using a flow cytometry gating strategy^17^. Besides, platelets can also release granules or microparticles to regulate innate and adaptive immunity. We also confirmed increased PMP release in patients with CKD, and found that PMP levels correlated positively with CD8^+^ T cell senescence. Although we demonstrated that CD8^+^ T cells can internalize PMPs in vivo, consistent with previous findings, direct in vivo evidence that PMPs induce CD8^+^ T cell senescence is still lacking and warrants further investigation^18,25,38^. Of note, recent studies indicate that platelet-releasing particles are closely associated with cellular senescence, but their performance is inconsistent in different milieu^39,40^. The release of platelet factor 4 by platelets in young mice alleviates cognitive impairment in older mice, whereas the release of miR-146 is upregulated in the hippocampus and frontal cortex of patients with Alzheimer disease. Given that platelets have fully functional miRNA machinery, we isolated PMPs and performed miRNA profiling to identify the leading cause of CD8^+^ T cell senescence. Here, we detected that miR-550a-5p is the most significantly upregulated miRNA in PMPs of patients with CKD and that it accelerates CD8^+^ T cell senescence. In addition, the upregulation of miR-550a-5p can also promote the proliferation and migration of cancer cells^41^. Taken together, it is plausible that miR-550a-5p may be implicated in the proliferation and migration of cancer cells via mediating CD8^+^ T cell senescence and immunosuppression. On the contrary, treating CD8^+^ T cells with a miR-550a-5p inhibitor effectively attenuates cell senescence when co-cultured with platelets from patients with CKD. Moreover, we found that the uraemic milieu drives miR-550a-5p upregulation in PMPs, although the responsible toxins remain unknown and future studies using defined uraemic toxins or toxin-depletion approaches are needed to pinpoint the precise upstream inducer. Interestingly, aspirin, an antiplatelet agent, also exhibits anti-ageing properties via antioxidant, proteostatic and immunometabolic mechanisms^42–44^. Hence, it is conceivable that antiplatelets may serve a promising strategy for alleviating CD8^+^ T cell senescence.

Mitochondrial dysfunction and associated ROS production have also been found in ageing and many ageing-associated pathological conditions, whereas the impairment of mitochondrial function and morphology also governs the senescent phenotype^26^. In our study, we observe that, after co-culture with platelets of patients with CKD, mitochondrial disorders in CD8^+^ T cells preceded the appearance of senescence phenotypes, whereas NAC treatment significantly rescued these effects, indicating that platelets of CKD may induce CD8^+^ T cell senescence by mediating mitochondrial dysfunction. In order to characterize the molecular mechanisms by which miR-550a-5p regulates CD8^+^ T cell senescence, we identify to our knowledge for the first time PMPCB as a target gene of miR-550a-5p using bioinformatics and dual luciferase reporter analyses. PMPCB is the catalytic subunit of the mitochondrial processing peptidase, which identifies and cleaves mitochondrial importing signals necessary for the proper transport of proteins into the mitochondria, and thus facilitates their maturation. A decrease or mutation in PMPCB levels would induce dysfunction of mitochondrial processing peptidase, thereby leading to accumulation of processing intermediates such as frataxin^45^. Meanwhile, PMPCB has been shown to play a key role in regulating oxidant stress and apoptosis mediated by mitochondrial dysfunction^46,47^. Here, knockdown of PMPCB in CD8^+^ T cells exhibits similar effects, including decreased mitochondrial membrane potential, impaired mitochondrial respiratory function and increased ROS production. However, the specific mechanisms by which PMPCB deficiency induces mitochondrial dysfunction are not fully elucidated. Further investigations reveal that decreased PMPCB impairs the maturation of the pro-NDUFS8 precursor protein, leading to a reduced NAD^+^/NADH ratio. NDUFS8 is a nuclear-encoded core subunit of human mitochondrial Complex I, and NDUFS8 deficiency displays an apparent reduction in NAD^+^/NADH ratio and a decrease in mitochondrial membrane potential^48^. Crucially, intracellular reduced NAD^+^ levels are intimately linked to ageing^49^, whereas NMN treatment effectively attenuated mitochondrial dysfunction and cellular senescence in CD8^+^ T cells. Hence, our findings suggest that therapeutic intervention of the PMPCB–NDUFS8 axis opens up new opportunities to promote CD8^+^ T cell senescence and immune function.

In summary, these findings demonstrate that platelet-derived miR-550a-5p is responsible for CD8^+^ T cell senescence via the PMPCB–NDUFS8 axis in CKD. This study provides novel insights into PMP-mediated immune–platelet interactions and a potential strategy for preventing CD8^+^ T cell senescence in CKD, whereas their effects on other cell types remain to be explored.

## Supplementary information

Supplementary Information

MicroRNA profile

## Data Availability

Data from this study are available upon reasonable request to the corresponding author.
