# Supplementary material for: Platelet-derived microparticles induce CD8+ T cell senescence in chronic kidney disease via the transfer of microRNA-550a-5p
Source: Exp Mol Med. 2026 Oct 16;58(10):3593–608. doi: 10.1038/s12276-026-01876-z (PMC13737140; doi:10.1038/s12276-026-01876-z)
Supplement: Supplementary file 1 [file 12276_2026_1876_MOESM1_ESM.pdf]

Supplementary Information

1. Supplementary Tables

2. Supplementary Figures

Supplementary Tables

Supplementary Table 1. CMV serostatus of the screened population.

Characteristic	HC	CKD stage 3-5	Total
Candidates	88	197	285
CMV seropositive status, n (%)			
Positive (≥14.0 U/mL)	42 (47.7%)	119 (60.4%)	161 (56.5%)
Equivocal (≥12.0 to <14.0 U/mL)	1 (1.1%)	3 (1.5%)	4 (1.4%)
Negative (<12.0 U/mL)	45 (51.1%)	75 (38.1)	120 (42.1%)
Final cohort	45 (51.1%)	75 (38.1)	120 (42.1%)

CMV IgG was determined by iFlash CMV IgG assay kit (YHLO, Shenzhen, China).

Abbreviations: HC, healthy control; CKD, chronic kidney disease; CMV, cytomegalovirus.

Supplementary Table 2 The basic characteristic of the enrolled volunteers

Variables	HC (N = 45)	CKD stage 3-5 (N = 75)	P-value
Age, years	35.2 ± 5.8	35.6 ± 5.7	.740
Male, n (%)	21 (46.7%)	46 (61.3%)	0.169
Current smoker, n (%)	8 (17.8%)	23 (30.7%)	0.178
Platelet counts, $\times 10^9$/L	252.4 ± 67.1	212.5 ± 69.9	0.003
Plateletcrit, %	0.3 ± 0.1	0.2 ± 0.1	0.002
Mean platelet volume, fl	10.5 ± 1.3	10.7 ± 1.2	0.448
Platelet-large cell ratio, %	27.5 ± 8.1	30.57 ± 9.01	0.042
Platelet distribution width, %	13.2 ± 2.7	13.3 ± 3.3	0.935
Lymphocyte count, $\times 10^9$/L	2.2 ± 0.9	1.6 ± 0.6	<.001
Lymphocyte percentage, %	28.1 ± 8.3	23.0 ± 7.6	<.001
Uric acid, μmol/L	344.4 ± 64.3	453.6 ± 138.0	<.001
CRP, mg/L	2.8 ± 1.7	6.4 ± 4.0	<.001
Urea, mmol/L	5.1 ± 1.6	18.3 ± 9.7	<.001
SCr, μmol/L	66.6 ± 16.0	509.6 ± 302.3	<.001
eGFR, mL/min/1.73 m^2	113.6 ± 12.5	18.6 ± 16.2	<.001
Calcium, mmol/L	2.3 ± 0.2	2.1 ± 0.3	<.001
Phosphorus, mmol/L	1.1 ± 0.2	1.5 ± 0.5	<.001
CMV IgG, U/mL	2.5 ± 0.8	2.7 ± 1.2	0.197
CMV seropositive, n (%)	0 (0%)	0 (0%)	

CMV IgG was determined by iFlash CMV IgG assay kit (YHLO, Shenzhen, China), values of <12.0 U/mL were negative. Abbreviations: CKD, chronic kidney disease; CRP, C-reactive protein; SCr, serum creatinine; eGFR, estimated glomerular filtration rate; CMV, cytomegalovirus.

Supplementary Table 3. The sequence sets for agomir.

agomir (human)	Primer Sequence (5'-3')	
hsa-miR-550a-5p agomir	Sense (5'-3')	AGUGCCUGAGGGAGUAAGAGCCC
	Antisense (5'-3')	GGGCUCUUACUCCCUCAGGCACU
mimics nc	Sense (5'-3')	UCACAACCUCCUAGAAAGAGUAGA
	Antisense (5'-3')	UCUACUCUUUCUAGGAGGUUGUGA

Supplementary Table 4. List of antibodies used in this study.

Antibodies	Source	Clone	Assay
PE Anti-Human CD3	Thermo Fisher	MEM-57	FC
APC Anti-Human CD4	Biolegend	OKT4	FC
BV421 Anti-Human CD8	BD	RPA-T8	FC
PE-CYN7 Anti-Human CD57	Thermo Fisher	TB01	FC
BV605 Anti-Human PD-1	BD	EH12	FC
EF450 Anti-Human CD28	Invitrogen	CD28.2	FC
FITC Anti-Human/Mouse Phospho-H2A.X (Ser139)	BioLegend	2F3	FC/WB/IF
Anti-human/mouse p21 antibody	abcam	EPR18021	FC/WB/IF
Anti-Human/Mouse PMPCB antibody	Thermo Fisher	9F13E4	WB/IF/IP
Anti-Human/Mouse NDUFS8 antibody	abcam	EPR11892	WB/IP
APC-CY7 Anti-Mouse CD3	Biolegend	17A2	FC
PE-Cy7 Anti-Mouse CD8α	Abmart	53-6.7	FC
BV605 Anti-Mouse CD44	BD	IM7	FC
PE Anti-Mouse CD153	Biolegend	RM153	FC
FITC Anti-Human CD41	Biolegend	HIP8	FC
EF450 Anti-Human CD45	Invitrogen	HI30	FC
APC Anti-Human CD61	Biolegend	VI-PL2	FC
APC Anti-Human CD62P	Thermo Fisher	Psel.KO2.3	FC
FITC Anti-Mouse CD41	Biolegend	MWReg30	FC
β-Actin antibody	Cell Signaling Technology	13E5	WB
Anti-Human CD63	Beyotime	Cat# AF0605	WB
Anti-Human CD9	Beyotime	Cat# AF0611	WB
Anti-Human CDTSG101	Beyotime	Cat# AF0608	WB
COX IV antibody	Servicebio	Cat# GB15250	WB/IF
HRP labeled goat anti-Rabbit IgG (H+L)	Abcam	Cat# 205718	WB
HRP labeled goat anti-Mouse IgG (H+L)	Abcam	Cat# 205719	WB

Supplementary Table 5. The sequence sets for siRNA

siRNA (human)	Primer Sequence (5'-3')	
siPMPCB-si-1	Sense (5'-3')	CGAAAAUGAGAAGAACAAUGGdTdT
	Antisense (5'-3')	CCAUUGUUCUUCUCAUUUUCGdTdT
siPMPCB-si-2	Sense (5'-3')	CAUUUCGGUGACUCUUUAUGCdTdT
	Antisense (5'-3')	GCAUAAAGAGUCACCGAAAUGdTdT
siPMPCB-si-3	Sense (5'-3')	GAAGUAUGUACCAAAUACAUUdTdT
	Antisense (5'-3')	AAUGUAUUUGGUACAUACUUCdTdT
NC	Sense (5'-3')	UUCUCCGAACGUGUCACGUdTdT
	Antisense (5'-3')	ACGUGACACGUUCGGAGAAdTdT

Supplementary Table 6. The sequence sets for miRNA

Oligo (human)	Primer Sequence (5'-3')	
hsa-miR-550a-5p	Sense (5'-3')	AGUGCCUGAGGGAGUAAGAGCCC
mimics	Antisense (5'-3')	GGGCUCUUACUCCCUCAGGCACU
hsa-miR-597-3p	Sense (5'-3')	UGGUUCUCUUGUGGCUCAAGCGU
mimics	Antisense (5'-3')	ACGCUUGAGCCACAAGAGAACCA
hsa-miR-4488	Sense (5'-3')	AGGGGGCGGGCUCCGGCG
mimics	Antisense (5'-3')	CGCCGGAGCCCGCCCCCU
hsa-miR-6804-5p	Sense (5'-3')	UGAGGGUGUCAGCAGGUGACG
mimics	Antisense (5'-3')	CGUCACCUGCUGACACCCUCA
hsa-miR-4470	Sense (5'-3')	UGGCAAACGUGGAAGCCGAGA
mimics	Antisense (5'-3')	UCUCGGCUUCCACGUUUGCCA
hsa-miR-550a-5p	Sense (5'-3')	
inhibitor	Antisense (5'-3')	GGGCUCUUACUCCCUCAGGCACU
mimics nc	Sense (5'-3')	UCACAACCUCCUAGAAAGAGUAGA
	Antisense (5'-3')	UCUACUCUUUCUAGGAGGUUGUGA
inhibitor nc	Sense (5'-3')	
	Antisense (5'-3')	UCUACUCUUUCUAGGAGGUUGUGA

Supplementary Table 7. The primer sets for human qPCR

Gene (human)	Primer Sequence (5'-3')
h-miR-550a-5p RT stem loop	GTCGTATCCAGTGCAGGGTCCGAGGTATTCGCACTGGATACGACGGGCTC
miR-550a-5p F	TGCTGTTAGGTTGTCTTCA
miR-550a-5p R	CTATGTTTTGTCCAATTTCT
hsa-miR-6804-5p RT stem loop	GTCGTATCCAGTGCAGGGTCCGAGGTATTCGCACTGGATACGACCGTCAC
hsa-miR-6804-5p-F	CGCGTGAGGGTGTCAGCAG
hsa-miR-6804-5p-R	AGTGCAGGGTCCGAGGTATT
hsa-miR-4470 RT stem loop	GTCGTATCCAGTGCAGGGTCCGAGGTATTCGCACTGGATACGACTCTCGG
hsa-miR-4470-F	GCGTGGCAAACGTGGAAG
hsa-miR-4470-R	AGTGCAGGGTCCGAGGTATT
hsa-miR-597-3p RT stem loop	GTCGTATCCAGTGCAGGGTCCGAGGTATTCGCACTGGATACGACACGCTT
hsa-miR-597-3p-F	GCGTGGTTCTCTTGTGGCTC
hsa-miR-597-3p-R	AGTGCAGGGTCCGAGGTATT
hsa-miR-4488 RT stem loop	GTCGTATCCAGTGCAGGGTCCGAGGTATTCGCACTGGATACGACCGCCGG
hsa-miR-4488-F	CGCGAGGGGGCGGGCT
hsa-miR-4488-R	AGTGCAGGGTCCGAGGTATT
h-U6 RT stem loop	GTCGTATGCAGAGCAGGGTCCGAGGTATTCGCACTGCATACGACAAAATATGG
h-U6 F	CTCGCTTCGGCAGCACA
h-U6 R	AACGCTTCACGAATTTGCGT
PMPCB-F	TGGGGTTTCAGCGAGAGTCT
PMPCB-R	TGGGTAGCAGCCTGTGTACTT
GHITM-F	GGCCTCAGTATGTCAAGGATAGA
GHITM-R	GCGTTCTGCTGATTGCTATGG
SLC25A37-F	GATGGGGACAGCCGAGATG
SLC25A37-R	ACCGGGTACATGACCGAGT
BCL2L2-F	GCGGAGTTCACAGCTCTATAC
BCL2L2-R	AAAAGGCCCCTACAGTTACCA
NDUFS8-F	CCATCAACTACCCGTTCGAGA
NDUFS8-R	CCGCAGTAGATGCACTTGG
IFN-γ-F	TCGGTAACTGACTTGAATGTCCA
IFN-γ-R	TCGCTTCCCTGTTTTAGCTGC
TNF-α-F	CCTCTCTCTAATCAGCCCTCTG
TNF-α-R	GAGGACCTGGGAGTAGATGAG

Gene (human)	Primer Sequence (5'-3')
β-actin-F	CATGTACGTTGCTATCCAGGC
β-actin-R	CTCCTTAATGTCACGCACGAT

Supplementary Table 8. The primer sets for mouse qPCR

Gene (mouse)	Primer Sequence (5'-3')
mmu-IFN-γ-F	AGCAAGGCGAAAAAGGATGC
mmu-IFN-γ-R	TCATTGAATGCTTGGCGCTG
mmu-TNF-α-F	CCCTCACACTCACAAACCAC
mmu-TNF-α-R	ACAAGGTACAACCCATCGGC
mmu-β-actin-F	TGAGCTGCGTTTTACACCCT
mmu-β-actin-R	GCCTTCACCGTTCCAGTTTT

Supplementary Table 9. Predicted targets of miR-550-5p using TargetScan, miRDB and MitoCarta.

GENE			
NT5DC3	PRELID2	SLC25A37	GHITM
UNG	PMPCB	ALKBH1	WARS2
BCL2L2	MRPL44	CYP27B1	SUCLA2
RNASEH1	PDP2	ECHDC2	MTFMT
GTPBP10	NDUFAF4	HOGA1	

Supplementary Figures

Supplementary Fig. 1

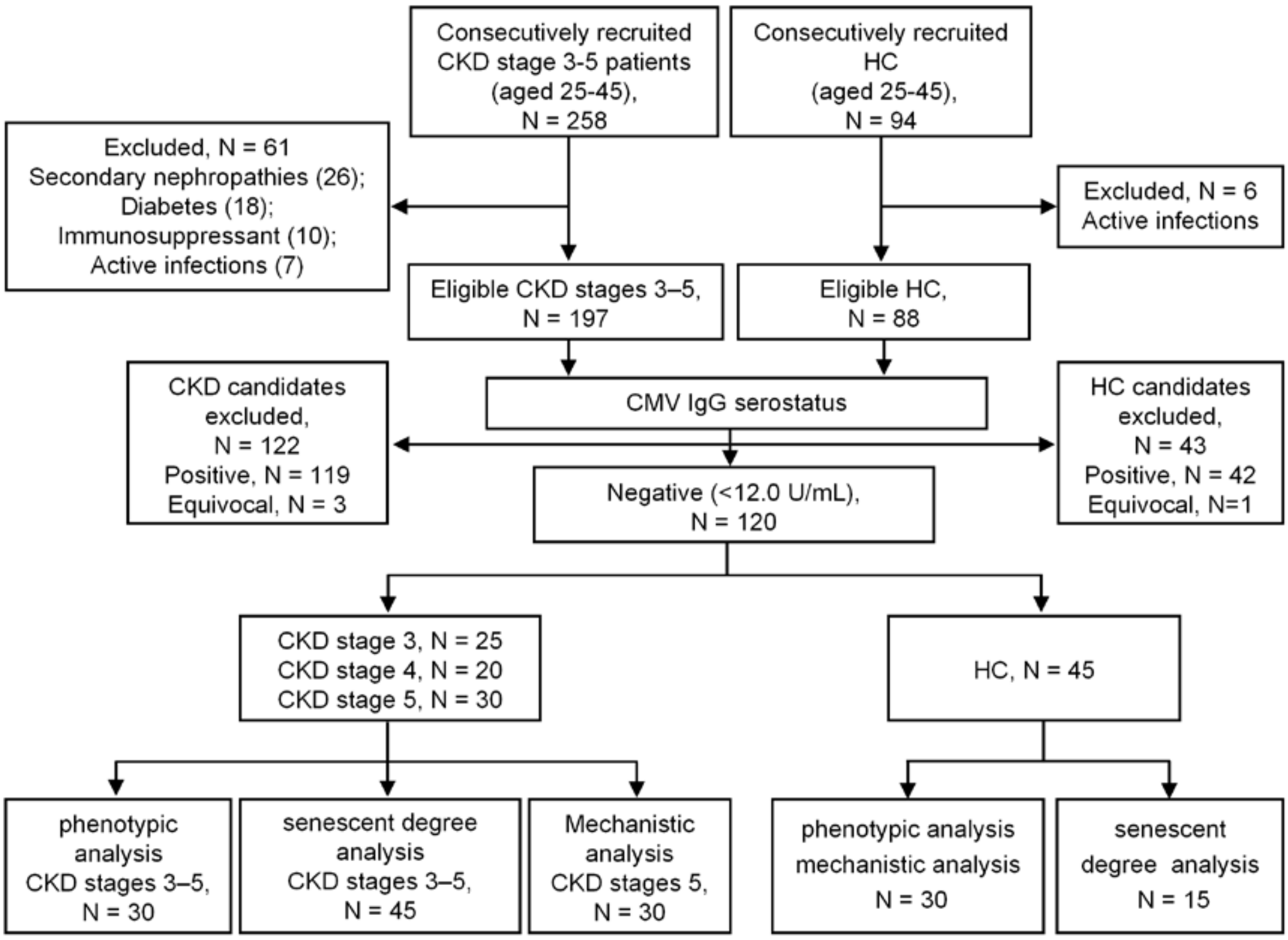

Supplementary Fig. 1. Flow diagram of participant screening, CMV IgG testing, enrollment, and allocation to subsequent analyses. CKD patients and healthy controls were consecutively screened according to the prespecified eligibility criteria. Candidates with positive or equivocal CMV IgG results were excluded. Mechanistic analyses were performed on a subset of participants from the phenotypic and senescent degree analysis cohorts. The same 30 healthy controls were used for the phenotypic and mechanistic analyses. Abbreviations: CKD, chronic kidney disease; CMV, cytomegalovirus; HC, healthy control.

Supplementary Fig. 2

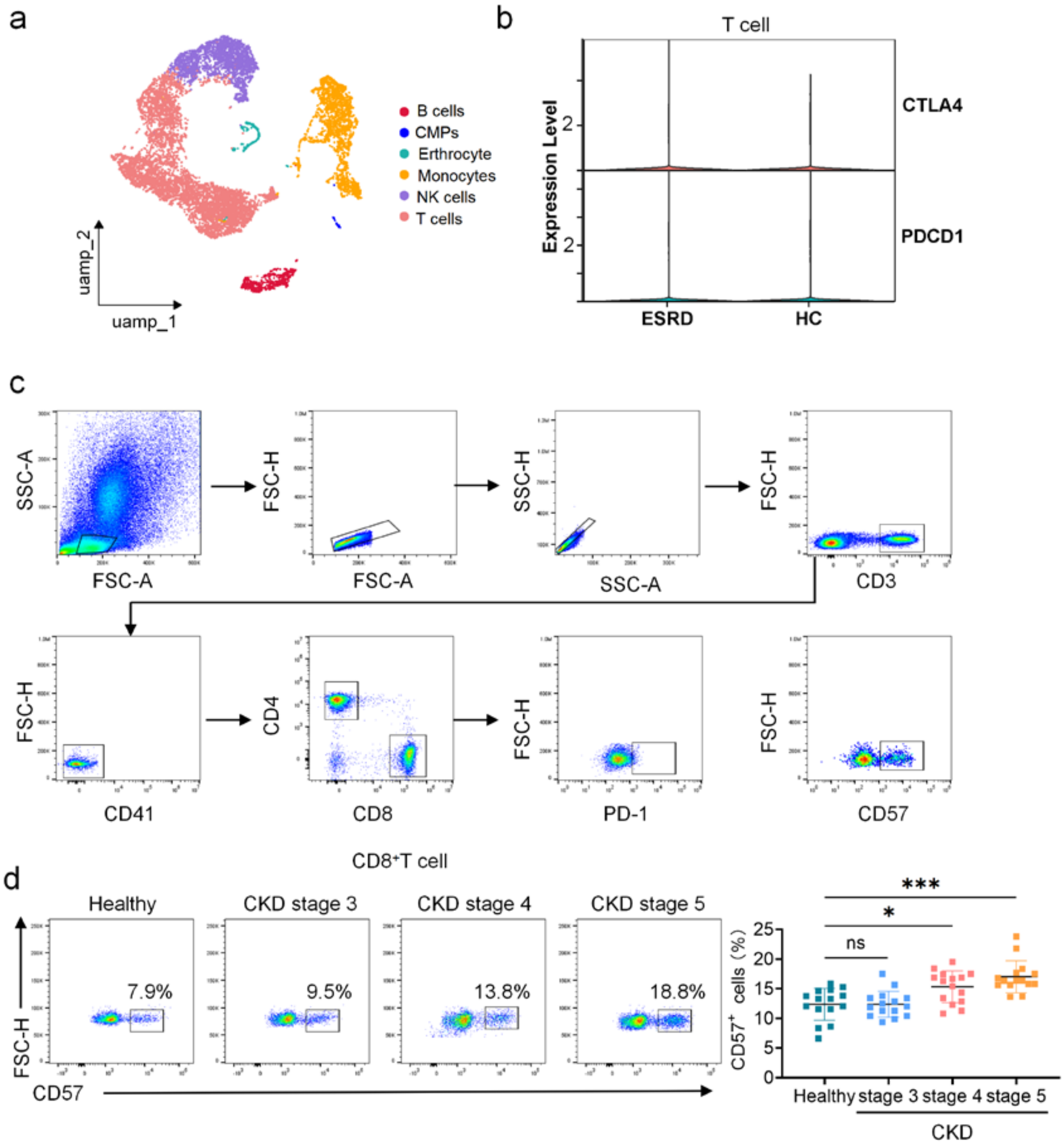

Supplementary Fig. 2. Premature senescence in CD8^{+} T cells progressively develop along with advancing CKD. (a) t-SNE projection of PBMCs from healthy controls and ESRD patients, colored by major cell clusters identified through scRNA-seq analysis. CMPs: Common Myeloid Progenitors. (b) Comparison of the PD-1 and CTLA4 expression in CD8^{+} T cells between the ESRD group and healthy group through scRNA-seq analysis. (c) Gating strategy of flow cytometry. (d) Flow cytometric analysis of the proportion of CD57^{+} cells in CD8^{+} T cells between healthy

controls and CKD patients with stage 3-5 (n = 15). Data are presented as mean ± SD and were analyzed by one-way ANOVA. ns: no significance. *$P < 0.05$, ***$P < 0.001$.

Supplementary Fig. 3

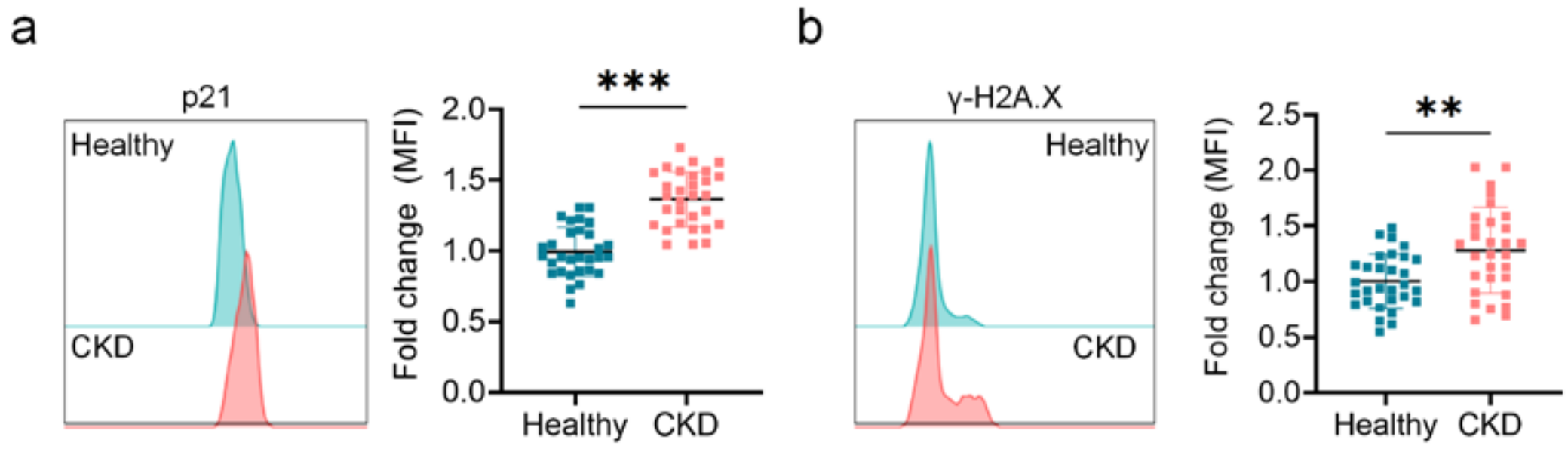

Supplementary Fig. 3. $CD8^+$ T cells exhibit upregulated senescence biomarkers in CKD. (a, b) Representative flow cytometry plots showing the expression levels of p21 expression (a) and γ-H2A.X level (b) in $CD8^+$ T cells of healthy controls and CKD patients (n = 30). Data are presented as mean ± SD and were analyzed unpaired Student's t-test. **P < 0.01, ***P < 0.001.

Supplementary Fig. 4

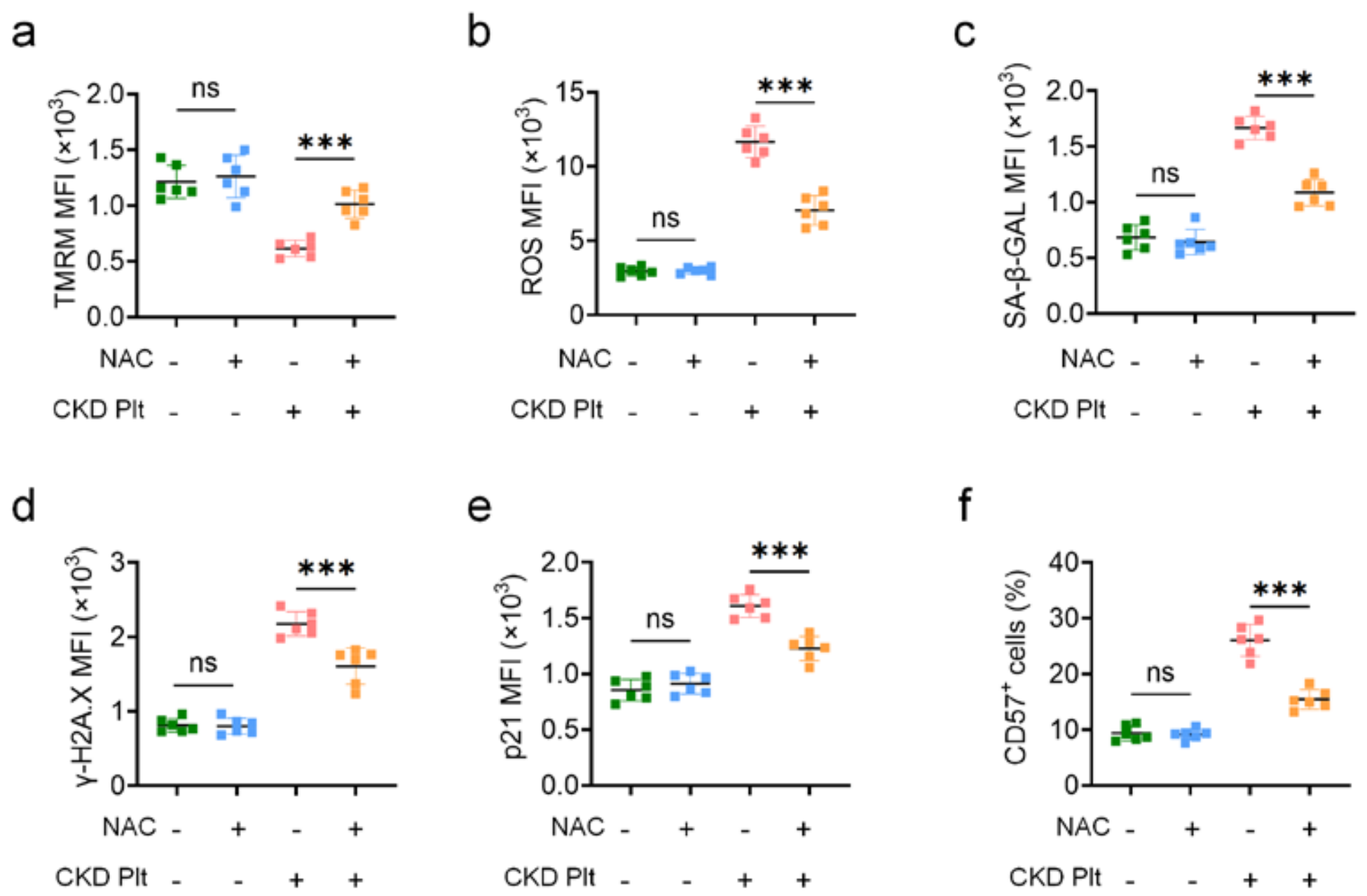

Supplementary Fig. 4. NAC significantly alleviates premature CD8⁺ T cell senescence induced by platelets from CKD patients. (a, b) Flow cytometric analysis of TMRM levels (a) and ROS levels (b) in CD8⁺ T cells on day 3 of co-culture, with or without supplementation of 5 mM NAC. (c-f) Flow cytometric analysis of SA-β-Gal activity (c), γ-H2AX level (d), p21 expression (e) and proportion of CD57⁺ cells (f) in CD8⁺ T cell on day 5 of co-culture, with or without supplementation of 5 mM NAC (n = 6). Data are presented as mean ± SD and were analyzed by one-way ANOVA. ***P < 0.001.

Supplementary Fig. 5

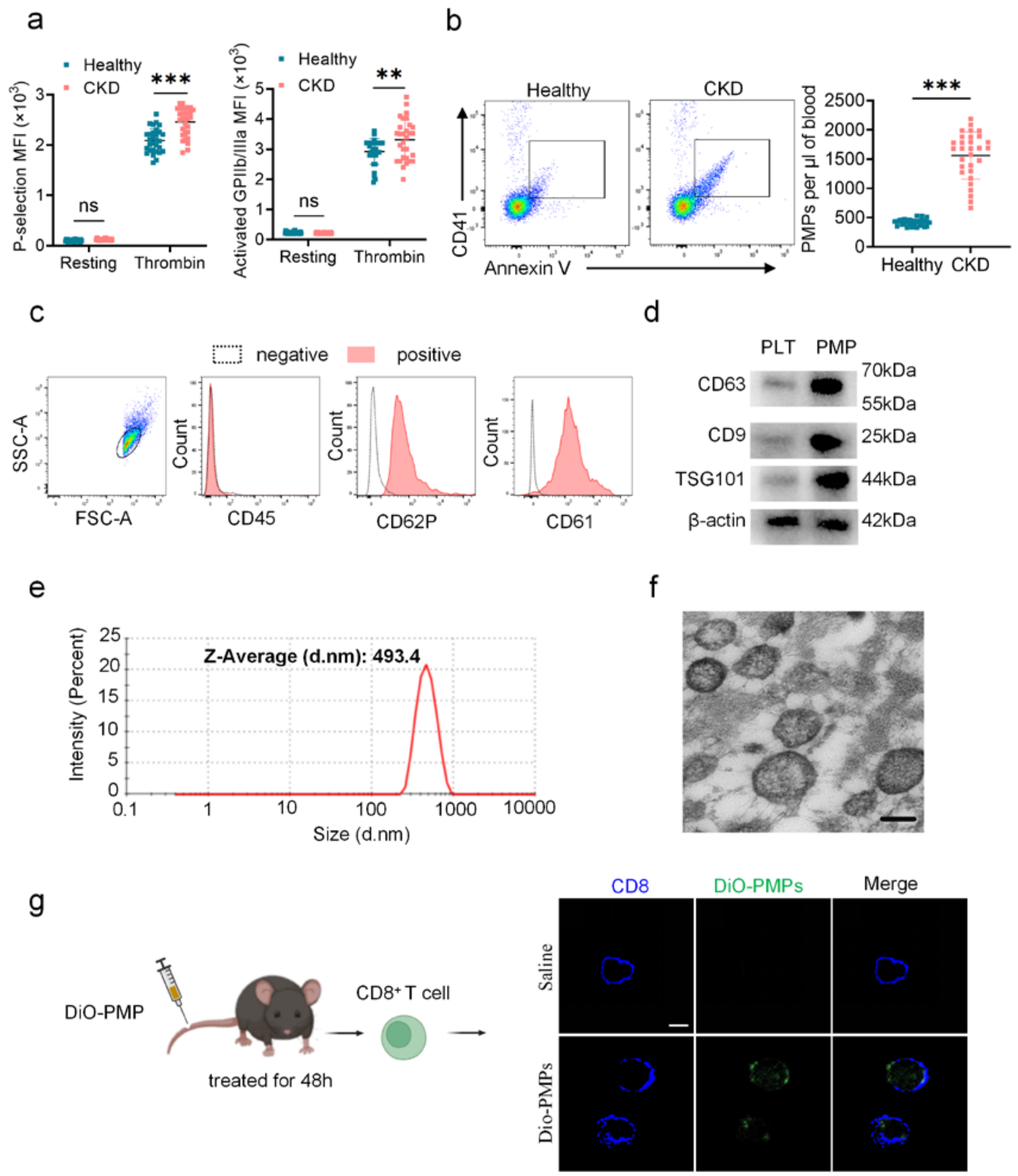

Supplementary Fig. 5. PMPs derived from CKD patients exhibit hyperactivation and characteristic vesicular features. (a) Flow cytometric analysis of P-selectin and activated GPIIb/IIIa expression in platelets from healthy controls or CKD patients, either untreated or treated with 0.1 U/mL thrombin (n = 30 for each analysis). (b) Flow cytometric quantification of PMP concentration in whole blood, expressed as

the number of PMPs per μL of whole blood from healthy controls or CKD patients (n = 30). (c) Representative flow cytometry plots showing the expression of CD45, CD61, CD62P in PMPs. (d) Western blot analysis of CD63, CD9, TSG101 expression in isolated PMPs and platelets. (e) Particle size analysis of isolated PMPs. (f) Representative transmission electron microscopy images of isolated PMPs. Scale bar, 200 nm. (g) Schematic diagram and representative immunofluorescence images illustrating the internalization of PMPs by $CD8^+$ T cells in vivo. PMPs were labeled with DiO (green), and of $CD8^+$ T cells were stained with anti-CD8 (blue). Scale bar, 5μm. Data are presented as mean ± SD and were analyzed by one-way ANOVA or 2-tailed, unpaired Student's t-test (a, b). ns: no significance. **P < 0.01, ***P < 0.001.

Supplementary Fig. 6

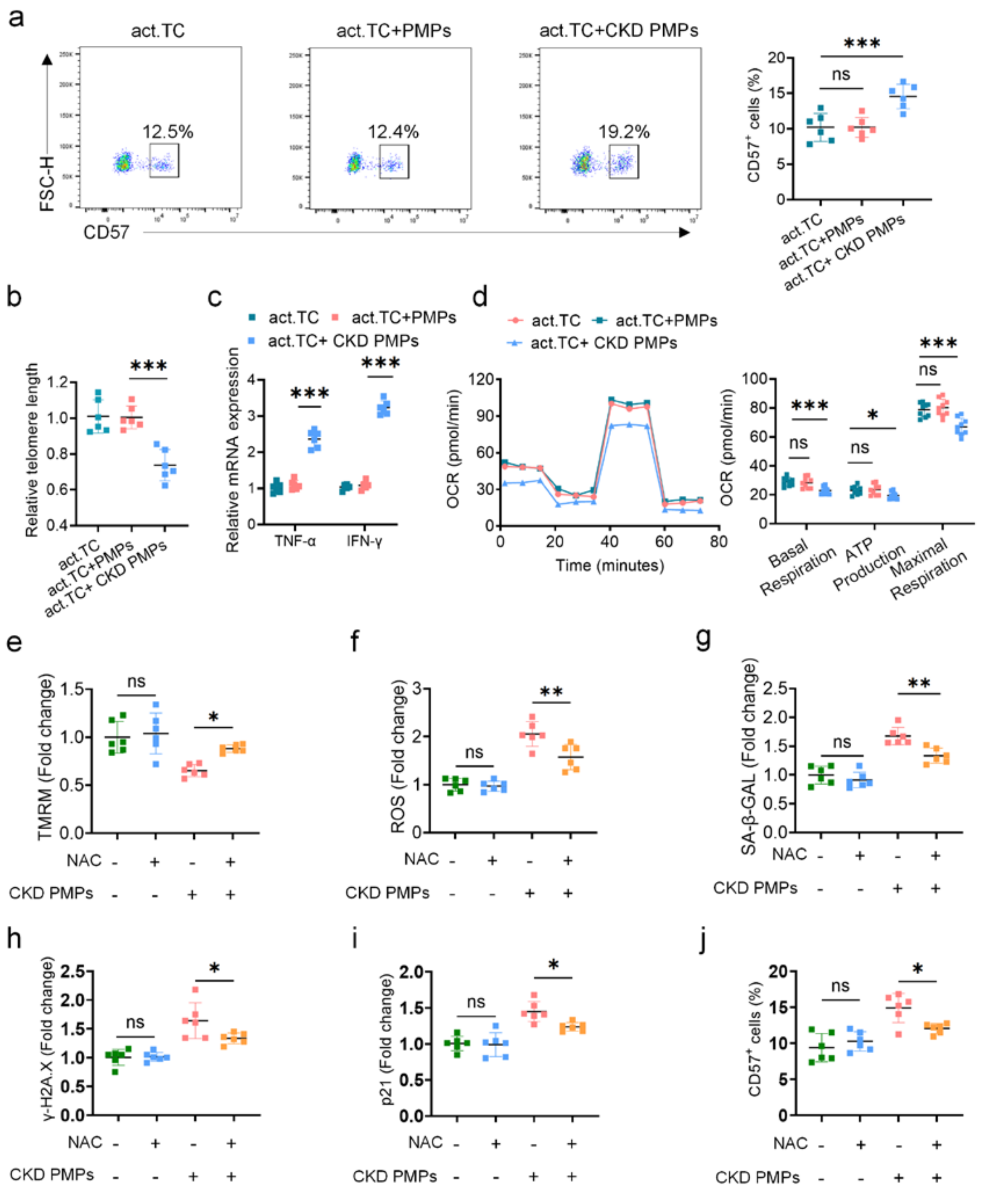

Supplementary Fig. 6. PMPs mediate mitochondrial dysfunction and cellular senescence in CD8$^+$ T cells. (a) Flow cytometric analysis of proportion of CD57$^+$ cells in CD8$^+$ T cells treated with PMPs from healthy controls or CKD patients (n = 6). (b) Relative telomere length in CD8$^+$ T cells treated with PMPs from healthy controls

or CKD patients (n = 6). (c) qPCR analysis of TNF-α and IFN-γ expression in $CD8^+$ T cells treated with PMPs from healthy controls or CKD patients (n = 6). (d) OCR in $CD8^+$ T cells treated with PMPs from healthy controls or CKD patients, with quantitation of basal respiration, ATP production, and maximal respiration (n = 9). (e-j) Flow cytometric analysis of TMRM levels (e), ROS levels (f), SA-β-Gal activity (g), γ-H2AX level (h), p21 expression (i) and proportion of $CD57^+$ (j) cells in $CD8^+$ T cell treated with PMPs from healthy controls or CKD patients, with or without supplementation of 5 mM NAC (n = 6). act. TC, activated $CD8^+$ T cells. Data are presented as mean ± SD and were analyzed by one-way ANOVA. ns: no significance. *P < 0.05, **P < 0.01, ***P < 0.001.

Supplementary Fig. 7

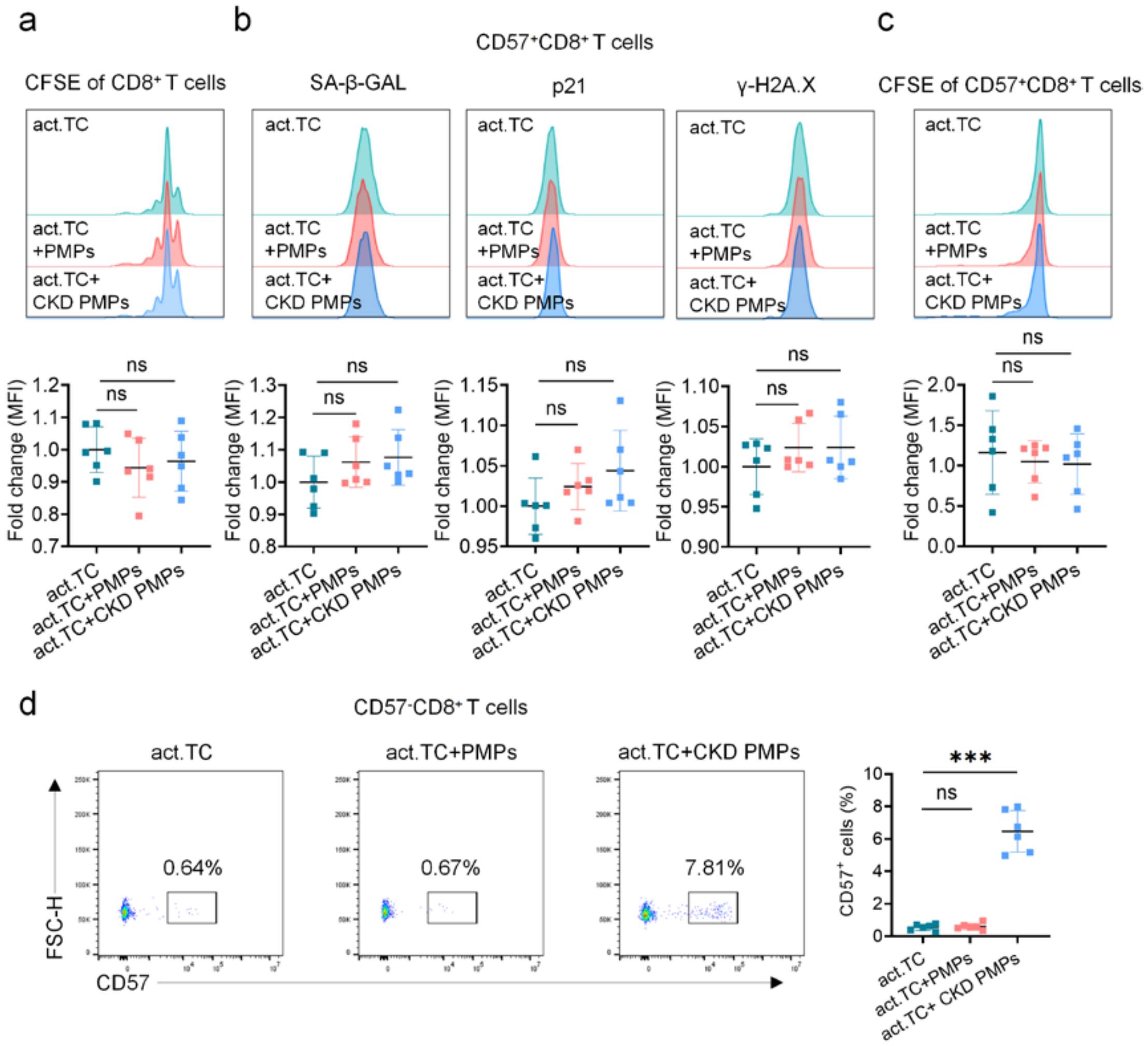

Supplementary Fig. 7. PMPs mediate CD57⁻ to CD57⁺ conversion in CD8⁺ T cells. (a) Flow cytometric analysis of CFSE in CD8⁺ T cells treated with PMPs from healthy controls or CKD patients (n = 6). (b) Flow cytometric analysis of SA-β-Gal activity, p21 expression, γ-H2AX level in CD57⁺CD8⁺ T cells treated with PMPs from healthy controls or CKD patients (n = 6). (c) Flow cytometric analysis of CFSE in CD57⁺CD8⁺ T cells treated with PMPs from healthy controls or CKD patients (n = 6). (d) Flow cytometric analysis of proportion of CD57⁺ cells in CD57⁻CD8⁺ T cells treated with PMPs from healthy controls or CKD patients (n = 6). act. TC, activated

$CD8^+$ T cells. Data are presented as mean ± SD and were analyzed by one-way ANOVA. ns: no significance. ***$P < 0.001$.

Supplementary Fig. 8

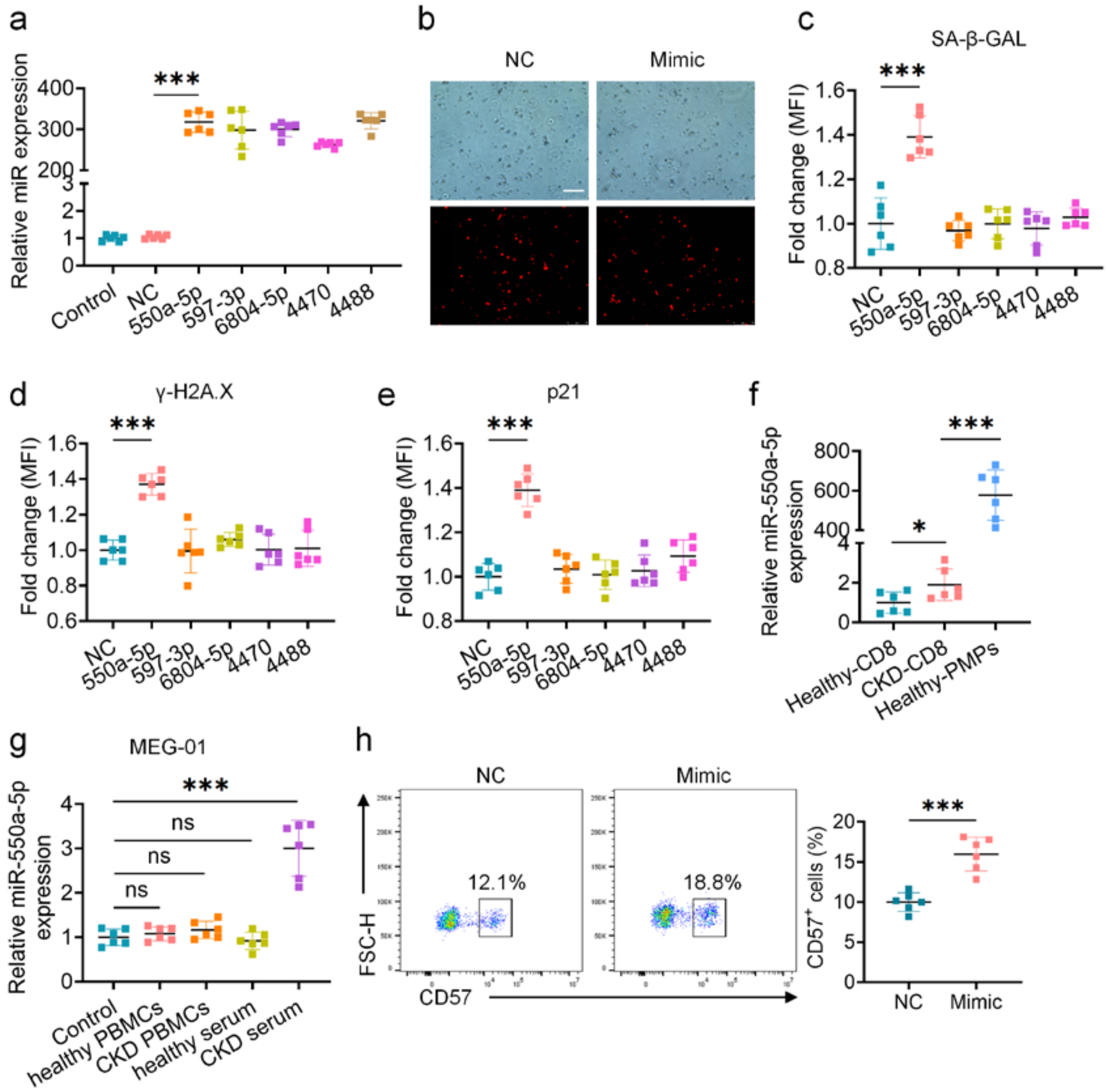

Supplementary Fig. 8. MiR-550a-5p mimic treatment increases mitochondrial dysfunction and cellular senescence in CD8⁺ T cell. (a) qPCR analysis of miRNAs expression in CD8$^+$ T cells treated with different miRNA mimic or control mimic (n = 6). (b) Representative fluorescence images of CD8$^+$ T cells after 24 hours post-transfection. Scale bar, 75 μm. (c-e) Flow cytometric analysis of SA-β-Gal activity (c), γ-H2AX level (d), p21 expression (e) in CD8$^+$ T cells treated with different miRNA mimic or control mimic (n = 6). (f) qPCR analysis of miR-550a-5p

expression in $CD8^+$ T cells of healthy controls and CKD patients and PMPs of healthy controls (n = 6). (g) qPCR analysis of miR-550a-5p expression in MEG-01 cells from different experimental groups (n = 6). (h) Flow cytometric analysis the proportion of $CD57^+$ cells in $CD8^+$ T cells treated with or without miR-550a-5p mimic (n = 6). Data are presented as mean ± SD and were analyzed by one-way ANOVA. ns: no significance. **P < 0.01, ***P < 0.001.

Supplementary Fig. 9

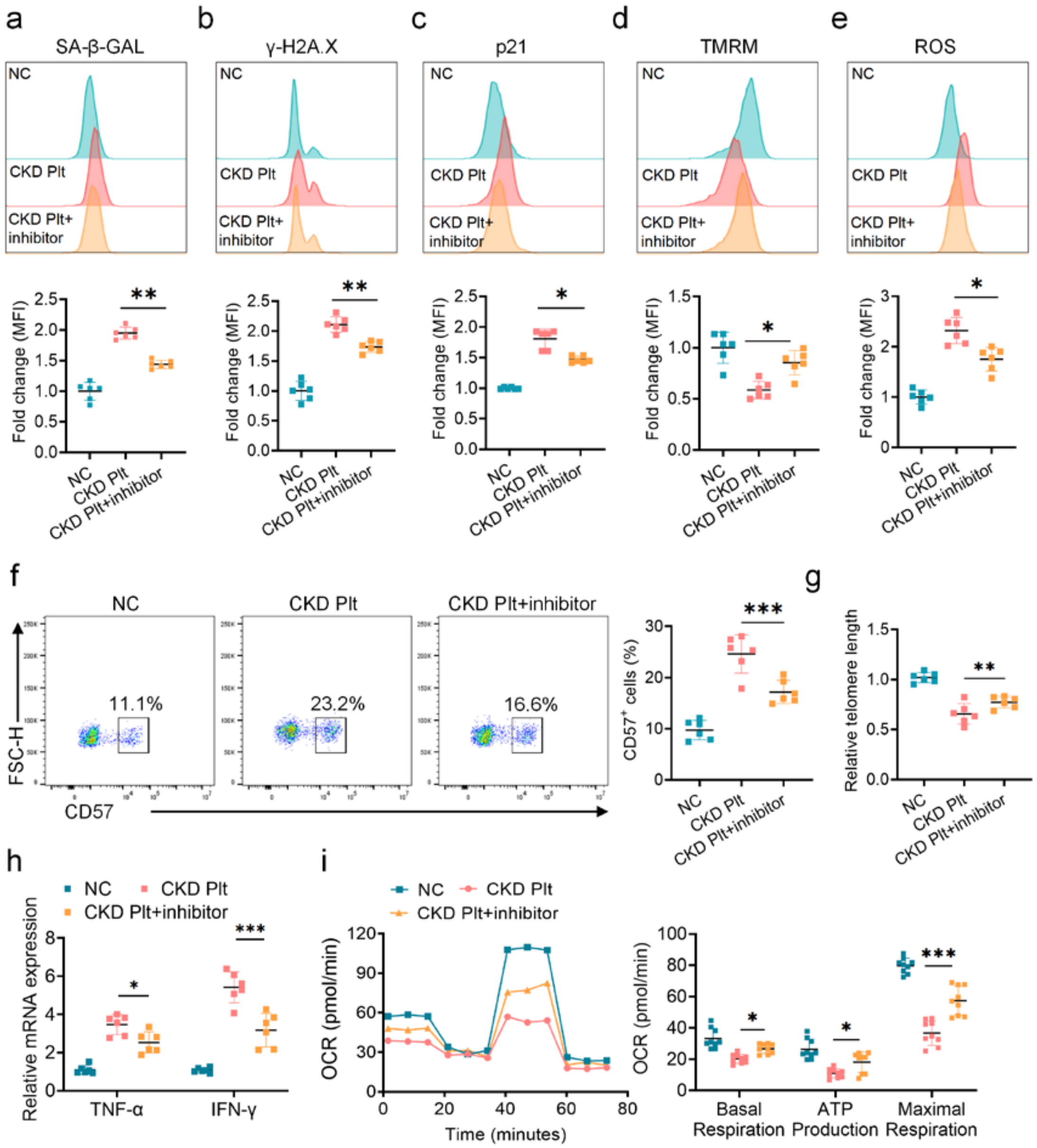

Supplementary Fig. 9. miR-550a-5p inhibitor alleviates mitochondrial dysfunction and cellular senescence in CD8⁺ T cells induced by platelets from CKD patients. (a-f) Flow cytometric analysis of SA-β-Gal activity (a), γ-H2AX level (b), p21 expression (c), TMRM levels (d), ROS levels (e) and proportion of CD57⁺ cells (f) in CD8⁺ T cells treated with miR-550a-5p inhibitor or negative control under

co-cultured system (n = 6). (g) Relative telomere length in $CD8^+$ T cells treated with miR-550a-5p inhibitor or negative control under co-cultured system (n = 6). (h) qPCR analysis of TNF-α and IFN-γ expression in $CD8^+$ T cells treated with miR-550a-5p inhibitor or negative control under co-cultured system (n = 6). (i) OCR in $CD8^+$ T cells treated with miR-550a-5p inhibitor or negative control under co-cultured system, with quantitation of basal respiration, ATP production, and maximal respiration (n = 9). Data are presented as mean ± SD and were analyzed by one-way ANOVA. *$P < 0.05$, **$P < 0.01$, ***$P < 0.001$.

Supplementary Fig. 10

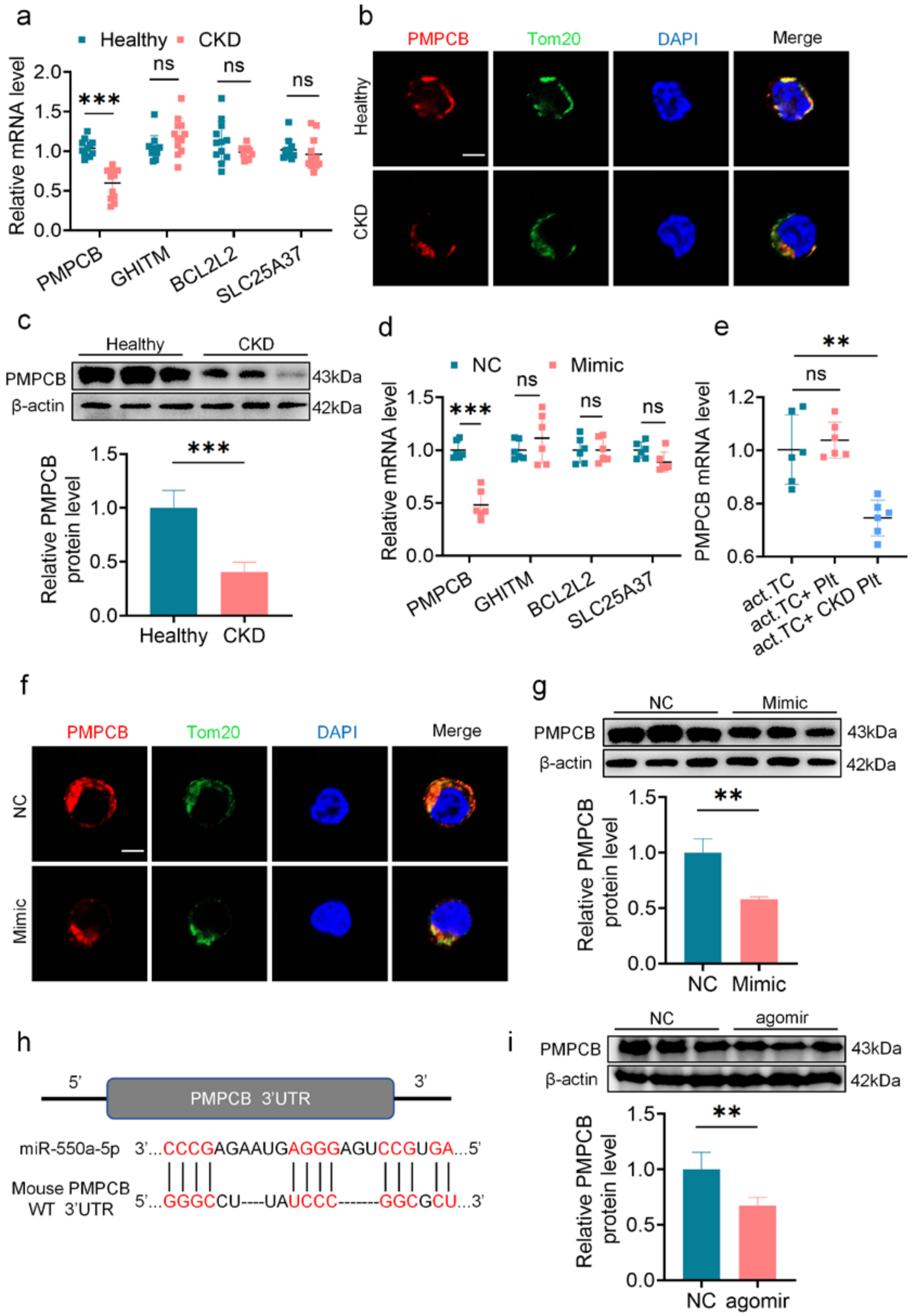

Supplementary Fig. 10. MiR-550a-5p regulates PMPCB expression via directly binding to its 3'UTR region. (a) qPCR analysis of expression levels of selected

potential target genes of miR-550a-5p in $CD8^+$ T cells of healthy controls and CKD patients (n = 12). (b) Representative immunofluorescence staining images showing PMPCB expression (red) in $CD8^+$ T cells of healthy controls and CKD patients. Mitochondria were labeled with Tom20 (green). Scale bar, 10 μm. (c) Western blot analysis of PMPCB expression in $CD8^+$ T cells of healthy controls and CKD patients (n = 3). (d) qPCR analysis of expression levels of selected potential target genes of miR-550a-5p in $CD8^+$ T cells treated with or without miR-550a-5p mimic (n = 6). (e) qPCR analysis of PMPCB expression in $CD8^+$ T cells co-cultured with platelets from healthy controls or CKD patients (n = 6). (f) Representative immunofluorescence staining images showing PMPCB expression (red) in $CD8^+$ T cells treated with or without miR-550a-5p mimic. Mitochondria were labeled with Tom20 (green). Scale bar, 10 μm. (g) Western blot analysis of PMPCB protein levels in $CD8^+$ T cells treated with or without miR-550a-5p mimic (n = 3). (h) Schematic illustration of the putative miR-550a-5p binding sites within the 3′ untranslated region (UTR) of the mouse PMPCB gene. (i) Western blot analysis of the PMPCB protein levels in peripheral blood $CD8^+$ T cells of mouse treated with or without miR-550a-5p agomir (n = 3). act. TC, activated $CD8^+$ T cells. Data are presented as mean ± SD and were analyzed by 2-tailed, unpaired Student's t-test or one-way ANOVA (e). ns: no significance. $^{**}P < 0.01$, $^{***}P < 0.001$.

Supplementary Fig. 11

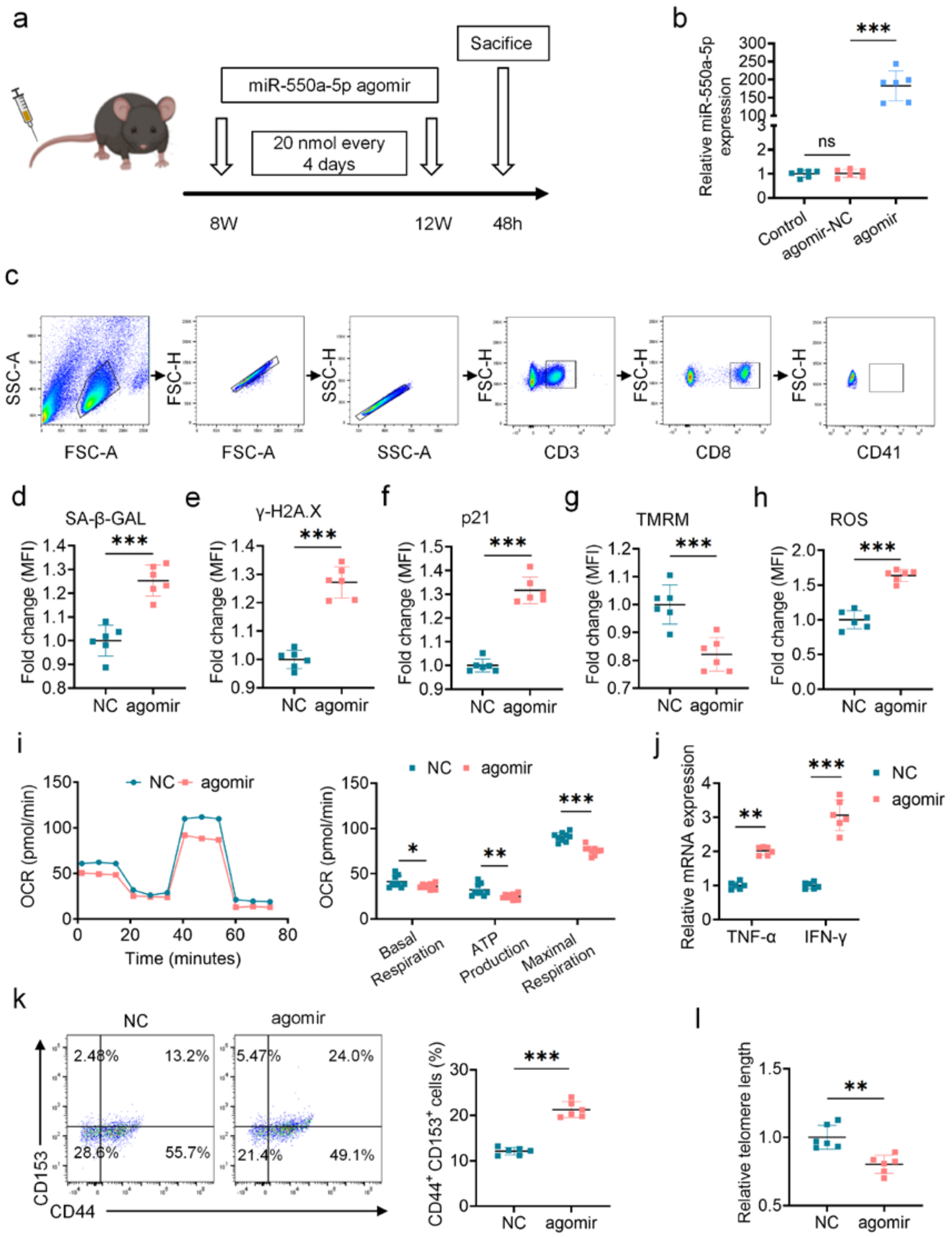

Supplementary Fig. 11. MiR-550a-5p agomir treatment increases mitochondrial dysfunction and cellular senescence of CD8$^+$ T cells in vivo. (a) Experimental outline. Peripheral blood CD8$^+$ T cells of mouse were harvested for functional assays. (b) qPCR analysis of miR-550a-5p expression in CD8$^+$ T cells of mouse treated with

miR-550a-5p agomir or negative control (n = 6). (c) Flow cytometric gating strategy for mouse lymphocyte subsets analysis. (d-h) Flow cytometric analysis of SA-β-Gal activity (d), γ-H2AX level (e), p21 expression (f), TMRM levels (g), and ROS levels (h) in $CD8^+$ T cells of mouse treated with miR-550a-5p agomir or negative control (n = 6). (i) OCR in $CD8^+$ T cells of mouse treated with miR-550a-5p agomir or negative control, with quantitation of basal respiration, ATP production, and maximal respiration (n = 9). (j) qPCR analysis of TNF-α and IFN-γ expression in $CD8^+$ T cells of mouse treated with miR-550a-5p agomir or negative control (n = 6). (k) Flow cytometric analysis of the proportion of $CD44^+CD153^+$ cells in $CD8^+$ T cells of mouse treated with miR-550a-5p agomir or negative control (n = 6). (l) Relative telomere length (L) in $CD8^+$ T cells of mouse treated with miR-550a-5p agomir or negative control (n = 6). Data are presented as mean ± SD and were analyzed by 2-tailed, unpaired Student's t-test or one-way ANOVA (b). ns: no significance. *P < 0.05, **P < 0.01, ***P < 0.001.

Supplementary Fig. 12

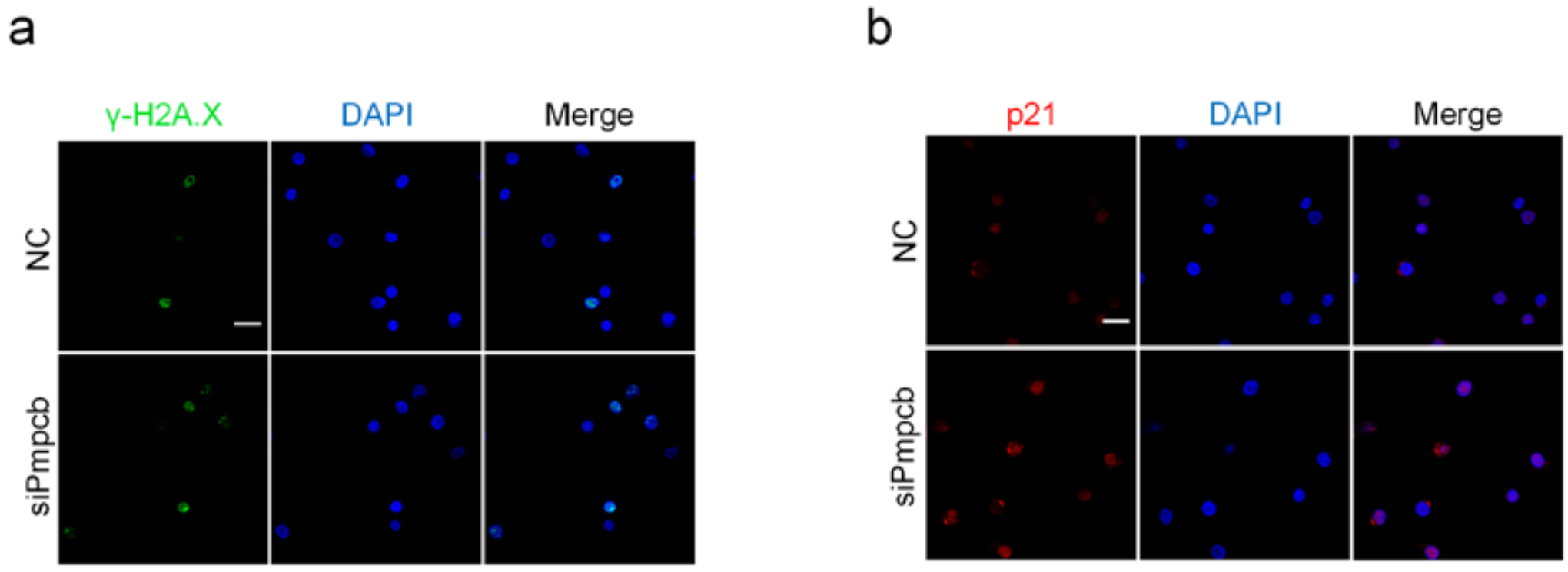

Supplementary Fig. 12. Knockown of PMPCB induces cellular senescence in CD8^{+} T cells. (a, b) Representative immunofluorescence staining images showing γ-H2A.X level (a) and p21 expression (b) in CD8^{+} T cells treated with PMPCB-specific siRNA or control siRNA. Scale bar, 20 μm.

Supplementary Fig. 13

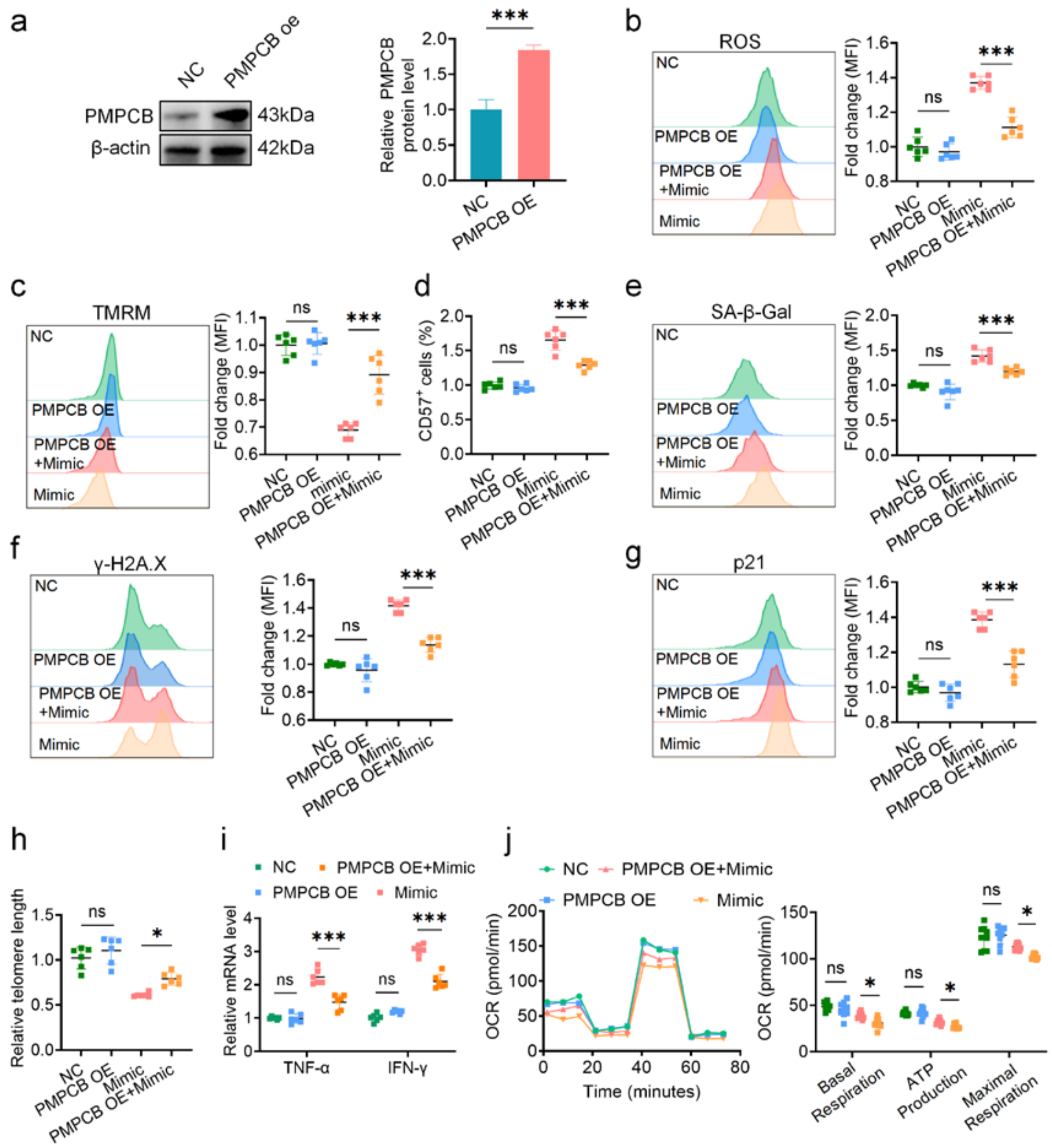

Supplementary Fig. 13. PMPCB overexpression improves miR-550a-5p-induced mitochondrial dysfunction and cellular senescence in $CD8^+$ T cells. (a) Western blot analysis of PMPCB protein levels in $CD8^+$ T cells transfected with PMPCB overexpression plasmid or control plasmid (n = 3). (b–g) Flow cytometric analysis of ROS levels (b), TMRM levles (c), proportion of $CD57^+$ cells (d), SA-β-Gal activity (e), γ-H2AX level (f), p21 expression (g) in $CD8^+$ T cells co-transfected with PMPCB

overexpression plasmid and miR-550a-5p mimic (n = 6). (h) Relative telomere length in $CD8^+$ T cells co-transfected with PMPCB overexpression plasmid and miR-550a-5p mimic (n = 6). (i) qPCR analysis of TNF-α and IFN-γ expression in $CD8^+$ T cells co-transfected with PMPCB overexpression plasmid and miR-550a-5p mimic (n = 6). (j) OCR in $CD8^+$ T cells co-transfected with PMPCB overexpression plasmid and miR-550a-5p mimic (n = 6), with quantitation of basal respiration, ATP production, and maximal respiration (n = 9). Data are presented as mean ± SD and were analyzed by one-way ANOVA or 2-tailed, unpaired Student's t-test (a). *$P < 0.05$, ***$P < 0.001$.

Supplementary Fig. 14

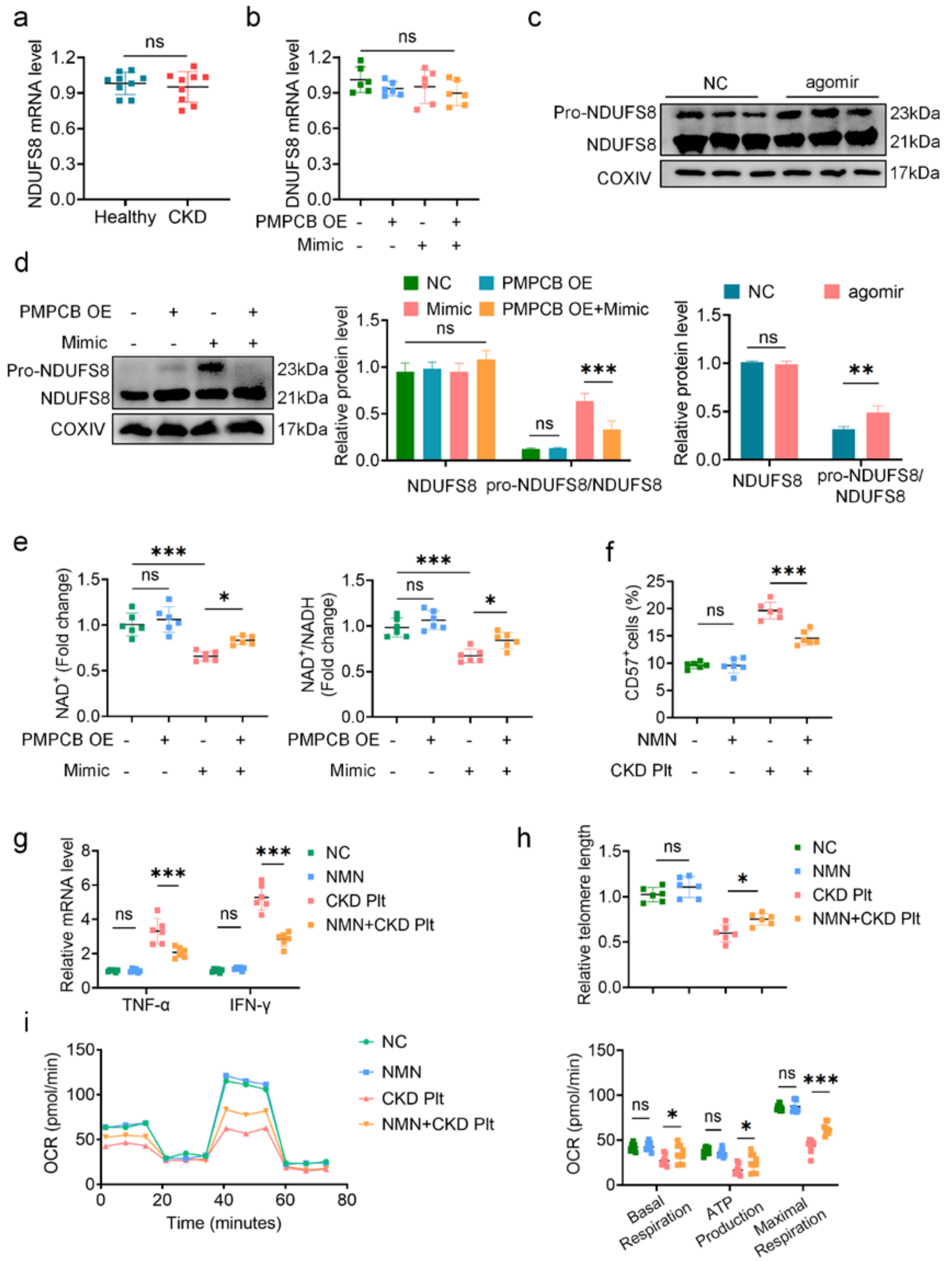

Supplementary Fig. 14. NMN counteracts platelets from CKD patients-induced and NDUFS8-mediated cellular senescence in $CD8^+$ T cells. (a) qPCR analysis of NDUFS8 expression in $CD8^+$ T cells of healthy controls and CKD patients (n = 9). (b)

qPCR analysis of NDUFS8 expression in $CD8^+$ T cells co-transfected with PMPCB overexpression plasmid and miR-550a-5p mimic (n = 6). (c) Western blot analysis of NDUFS8 expression in peripheral blood $CD8^+$ T cells of mouse treated with or without miR-550a-5p agomir (n = 3). (d) Western blot analysis of NDUFS8 expression co-transfected with PMPCB overexpression plasmid and miR-550a-5p mimic (n = 3). (e) NAD^+ content and NAD^+/NADH ratio in $CD8^+$ T cells co-transfected with PMPCB overexpression plasmid and miR-550a-5p mimic (n = 6). (f) Flow cytometric analysis the proportion of $CD57^+$ cells in $CD8^+$ T cells pretreated with or without 100 μM NMN under co-cultured system (n = 6). (g) qPCR analysis of TNF-α and IFN-γ expression in $CD8^+$ T cells pretreated with or without 100 μM NMN under co-cultured system (n = 6). (h) Relative telomere length in $CD8^+$ T cells pretreated with or without 100 μM NMN under co-cultured system (n = 6). (i) OCR in $CD8^+$ T cells pretreated with or without 100 μM NMN under co-cultured system, with quantitation of basal respiration, ATP production, and maximal respiration (n = 9). Data are presented as mean ± SD and were analyzed by ANOVA or 2-tailed, unpaired Student's t-test (a, c). ns: no significance. *P < 0.05, **P < 0.01, ***P < 0.001.

Supplementary Fig. 15

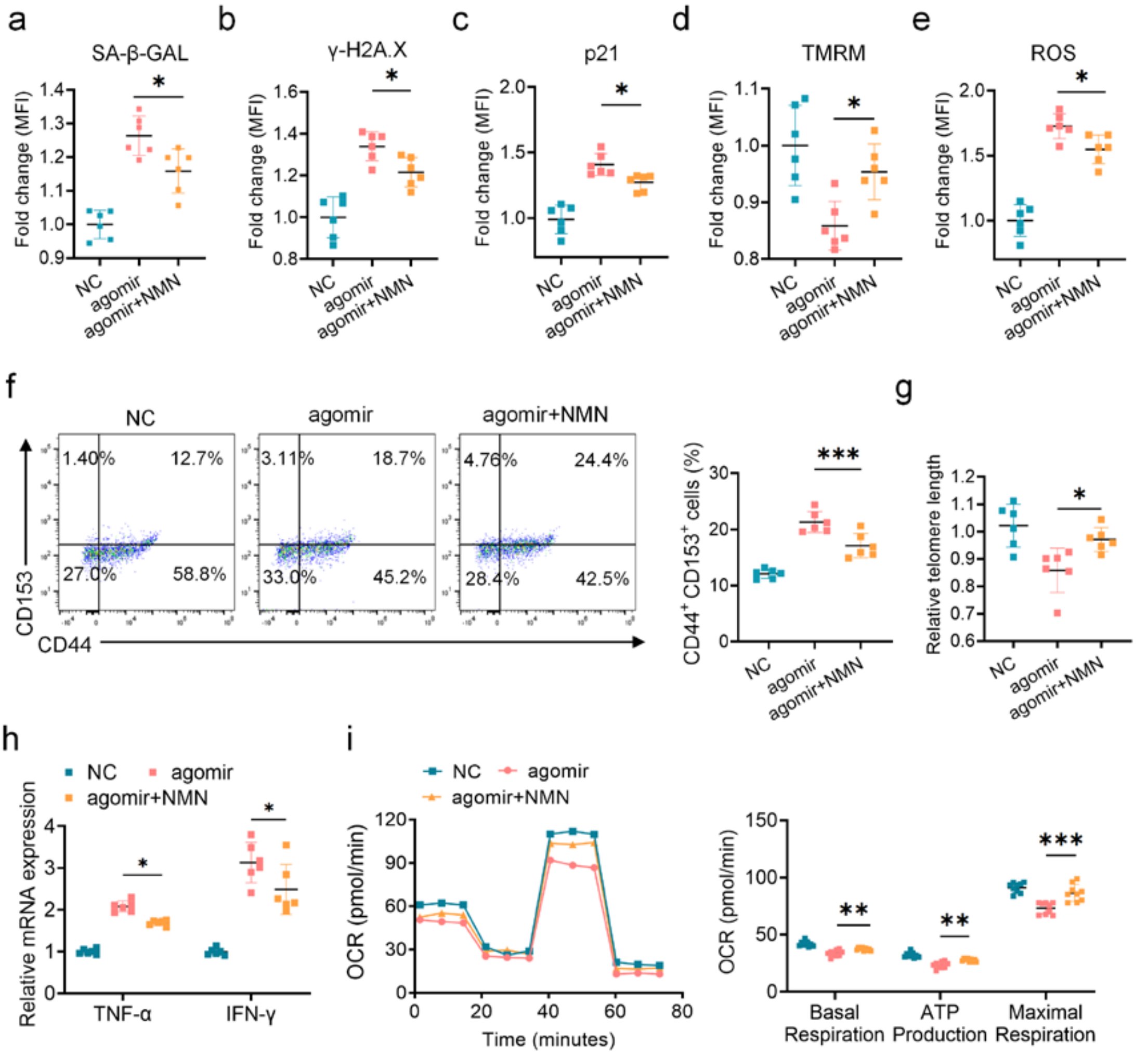

Supplementary Fig. 15. NMN counteracts MiR-550a-5p agomir-induced cellular senescence of CD8⁺ T cells in vivo. (a-f) Flow cytometric analysis of SA-β-Gal activity (a), γ-H2AX level (b), p21 expression (c), TMRM levels (d), and ROS levels (e), proportion of $CD44^{+}CD153^{+}$ cells (f) in peripheral blood $CD8^{+}$ T cells of mouse treated with or without miR-550a-5p agomir under NMN pretreatment (n=6). (g) Relative telomere length in peripheral blood $CD8^{+}$ T cells of mouse treated with or without miR-550a-5p agomir under NMN pretreatment (n = 6). (h) qPCR analysis of TNF-α and IFN-γ expression in peripheral blood $CD8^{+}$ T cells of mouse treated with

or without miR-550a-5p agomir under NMN pretreatment (n = 6). (i) OCR in peripheral blood $CD8^+$ T cells of mouse treated with or without miR-550a-5p agomir under NMN pretreatment, with quantitation of basal respiration, ATP production, and maximal respiration (n = 9). Data are presented as mean ± SD and were analyzed by one-way ANOVA. *P < 0.05, **P < 0.01, ***P < 0.001.
